# The Effects of Internet-Based Acceptance and Commitment Therapy on Process Measures: Systematic Review and Meta-analysis

**DOI:** 10.2196/39182

**Published:** 2022-08-30

**Authors:** Areum Han, Tae Hui Kim

**Affiliations:** 1 Department of Occupational Therapy University of Alabama at Birmingham Birmingham, AL United States; 2 Department of Psychiatry Yonsei University Wonju College of Medicine Wonju, Gangwon province Republic of Korea; 3 Department of Psychiatry Yonsei University Wonju Christian Hospital Wonju, Gangwon province Republic of Korea

**Keywords:** acceptance and commitment therapy, process measure, internet-based intervention, digital mental health, meta-analysis, mindfulness, systematic review

## Abstract

**Background:**

Acceptance and commitment therapy (ACT) is based on a psychological flexibility model that encompasses 6 processes: acceptance, cognitive defusion, self-as-context, being present, values, and committed action.

**Objective:**

This systematic review and meta-analysis of randomized controlled trials (RCTs) aimed to examine the effects of internet-based ACT (iACT) on process measures.

**Methods:**

A comprehensive search was conducted using 4 databases. The quality of the included RCTs was assessed using the Cochrane Collaboration Risk of Bias Tool. A random-effects or fixed-effects model was used. Subgroup analyses for each outcome were conducted according to the type of control group, use of therapist guidance, delivery modes, and use of targeted participants, when applicable.

**Results:**

A total of 34 RCTs met the inclusion criteria. This meta-analysis found that iACT had a medium effect on psychological flexibility and small effects on mindfulness, valued living, and cognitive defusion at the immediate posttest. In addition, iACT had a small effect on psychological flexibility at follow-up. The overall risk of bias across studies was unclear.

**Conclusions:**

Relatively few studies have compared the effects of iACT with active control groups and measured the effects on mindfulness, valued living, and cognitive defusion. These findings support the processes of change in iACT, which mental health practitioners can use to support the use of iACT.

## Introduction

### Background

Acceptance and commitment therapy (ACT), which is a type of mindfulness-based intervention, is an empirically supported transdiagnostic approach [1,2]. Mindfulness is defined as “awareness that arises through paying attention, on purpose, in the present moment, and nonjudgmentally” [3]. ACT aims to develop greater psychological flexibility, that is, the ability to face challenging experiences in an open, conscious manner and change one’s behaviors to participate in valued activities rather than avoiding or suppressing uncomfortable or painful experiences, emotions, and thoughts [1,2]. ACT is based on a psychological flexibility model involving 6 processes [2]. These six processes include (1) acceptance (ie, being open to unwanted thoughts and emotions as they are), (2) cognitive defusion (ie, stepping back from unhelpful thoughts and emotions to reduce their dominance over behaviors), (3) being present (ie, maintaining voluntary and flexible contact with the present moment), (4) observing self (ie, flexible self-conceptualization and perspective taking), (5) values (ie, identifying and connecting values to behaviors for a meaningful life), and (6) committed action (ie, establishing patterns of behaviors to live a meaningful life aligned with values) [2]. The first 2 processes (1 and 2) are conceptualized as *acceptance and mindfulness processes*, and the last 2 processes (5 and 6) are conceptualized as *commitment and behavior change processes*; the remaining 2 processes (3 and 4) are conceptualized to be shared by the *acceptance and mindfulness processes* and *commitment and behavior change processes* [4].

Studies have used ACT process measures to assess the effects of ACT on these process measures and to understand the processes of change [5,6]. Measuring psychological flexibility and its interrelated processes (eg, cognitive defusion, mindfulness, and valued living) has been emphasized to better understand why ACT works and how it directly affects these process measures [7]. Improvement of psychological flexibility and its interrelated processes is theorized to foster improvements in mental health outcomes, and this theory is supported by empirical studies to some extent [8]. For example, studies have found a significant predictive role of psychological flexibility in predicting mental health outcomes; negative associations of psychological flexibility with depressive symptoms, anxiety, and overall psychological distress; and positive associations of psychological flexibility with quality of life, emotional well-being, and resilience [9,10]. Studies have also shown negative relationships between cognitive defusion and psychological distress and a significant predictive role of cognitive fusion (ie, the opposite of cognitive defusion) for depressive symptoms, anxiety, distress, and lowered quality of life [11-14]. In addition, the literature suggests that personal values and mindfulness have a significant effect on psychological distress and quality of life [15,16].

Although a growing body of evidence shows that ACT can improve health and well-being outcomes in various populations [17,18], the synthesized evidence of ACT on process measures is lacking. For example, previous meta-analysis studies did not consider ACT process measures except for psychological flexibility, possibly because of the limited number of published studies and because previous meta-analysis studies often limited their review questions to specific populations [19]. Internet-based psychological interventions are easy to access and inexpensive; therefore, it is important to determine whether internet-based ACT (iACT) is an effective alternative option [20]. In particular, Brown et al [21] conducted a meta-analysis to measure the effects of iACT on outcomes related to mental health and well-being in any population, but none of the ACT process measures were addressed, possibly because of the limited number of included studies for meta-analyses of these outcomes at that time. In addition, Thompson et al [22] conducted a similar meta-analysis that involved only meta-analysis for psychological flexibility, with no meta-analyses for any other process measures.

### Objectives

This systematic review and meta-analysis aimed to assess the effects of iACT on different process measures (eg, psychological flexibility, valued living, mindfulness, and cognitive defusion) in any population. In addition, this meta-analysis aimed to conduct subgroup analyses for each outcome according to the type of control group to determine whether the effects of iACT differed when compared with active control groups provided with other comparable interventions and passive control groups provided with no intervention. In addition, other subgroup analyses related to the characteristics of the included studies may be possible and may provide useful information. For example, studies have found that iACT with therapist guidance showed larger effects on psychological flexibility compared with iACT without therapist guidance, and populations with psychological distress symptoms showed larger effects on mental health outcomes compared with nonclinical populations [22]. As studies have also found negative associations of psychological flexibility and cognitive defusion with psychological distress as well as a significant effect of personal values and mindfulness on psychological distress, studies that directly targeted people with psychological distress symptoms might show greater effects on ACT process measures [10-12,14-16]. Outcomes may differ based on how iACT was delivered (eg, web-based ACT modules, iACT accompanied by in-person ACT sessions, and videoconferencing ACT). Therefore, this study aimed to conduct additional subgroup analyses according to the use of therapist guidance, delivery modes, and the use of targeted participants with psychological distress symptoms, when applicable.

## Methods

The PRISMA (Preferred Reporting Items for Systematic Reviews and Meta-Analyses) guidelines [23] and the Cochrane Handbook for Systematic Reviews of Interventions (version 5.1.0) [24] were used as guides for conducting and reporting this systematic review and meta-analysis. This study was not preregistered.

### Inclusion and Exclusion Criteria

Studies were selected based on the following inclusion criteria: (1) the study must be a randomized controlled trial (RCT), (2) ACT must be delivered mainly on the web (ie, iACT), (3) the study must have pre-post test results in ACT process measures (eg, psychological flexibility, mindfulness, valued living, and cognitive defusion), (4) the study must compare iACT with a non-ACT condition or with a control condition, and (5) the study must be written in English. Studies were excluded if they compared ACT groups only with different delivery modes without any other comparison or control condition (eg, ACT delivered on the web vs ACT delivered in person).

### Search Strategy

Relevant articles were identified by searching 4 electronic databases from the date of inception of each database to June 5, 2021. The databases were PubMed (1966-2021), CINAHL (1981-2021), PsycINFO (1935-2021), and Scopus (1966-2021). Key search terms were combined to identify the relevant literature using keywords for iACT. To broaden the database search, keywords relevant to the outcomes were not entered as search terms. The full search strategies for all databases can be found in Multimedia Appendix 1. Articles were also searched manually using the reference lists of the identified articles and related article features in the databases.

### Data Extraction and Quality Assessment

The characteristics of the included RCTs, such as the country of origin, characteristics of participants, description of intervention and control groups, outcome measures, and results (ie, between-group differences with *P* values), were extracted into a table. The mean and SDs at each data collection time point and sample sizes of the intervention and control groups in the included studies were entered into a Microsoft Excel file. The methodological quality of the included RCTs was assessed using the Cochrane Collaboration Risk of Bias Tool [24]. The domains in the tool include random sequence generation, allocation concealment, blinding of participants and personnel, blinding of outcome assessment, incomplete outcome data, and selective reporting. Risk of bias in each of the domains was judged as *low risk* of bias, *high risk* of bias, or *unclear risk* of bias following the criteria provided in the Cochrane Collaboration’s handbook [24]. Summary assessments of the risk of bias within a study and across studies were also determined based on the handbook’s criteria [24]. One author with extensive experience in conducting systematic reviews and expertise in ACT completed the process for data extraction and quality assessment.

### Meta-analysis

Means, SDs, and sample sizes of intervention and control groups in the included studies were entered into RevMan (version 5.4; Cochrane Collaboration) for meta-analyses and pooled for each outcome at the immediate posttest and at follow-up. The *I*^2^ statistic was used to indicate statistical heterogeneity across studies, and *I*^2^>60% might indicate substantial heterogeneity [24]. The decision to use either a random-effects model or a fixed-effects model with the inverse variance method was determined using *I*^2^ statistic values for each outcome. In other words, a random-effects model was used when the *I*^2^ statistic for each variable was >60%; otherwise, a fixed-effects model was used. The standardized mean difference (SMD) with 95% CIs was used as a summary statistic for the size of the intervention effect to account for outcomes measured using different assessment tools [24]. SMDs<0.4 indicate a small effect, SMDs between 0.4 and 0.7 indicate a medium effect, and SMDs>0.7 indicate a large effect [24]. The mean difference, rather than SMD, was used when the studies used the same assessment tool [24]. Subgroup analyses for each outcome were performed according to the type of control group, if applicable, to see whether the effects of iACT differed compared with active control groups provided with other comparable interventions and compared with passive control groups provided with no intervention (ie, treatment as usual control groups and wait-list control groups). Additional subgroup analyses for each outcome were conducted according to the use of therapist guidance, delivery modes, and the use of targeted participants with psychological distress symptoms, when applicable. Funnel plot analysis was used to test for possible publication bias (ie, studies with positive findings are more likely to be published) [24]. A possible publication bias was suggested if the inverted funnel shape was asymmetrical [24].

## Results

### Selection of Studies

**Figure 1:**
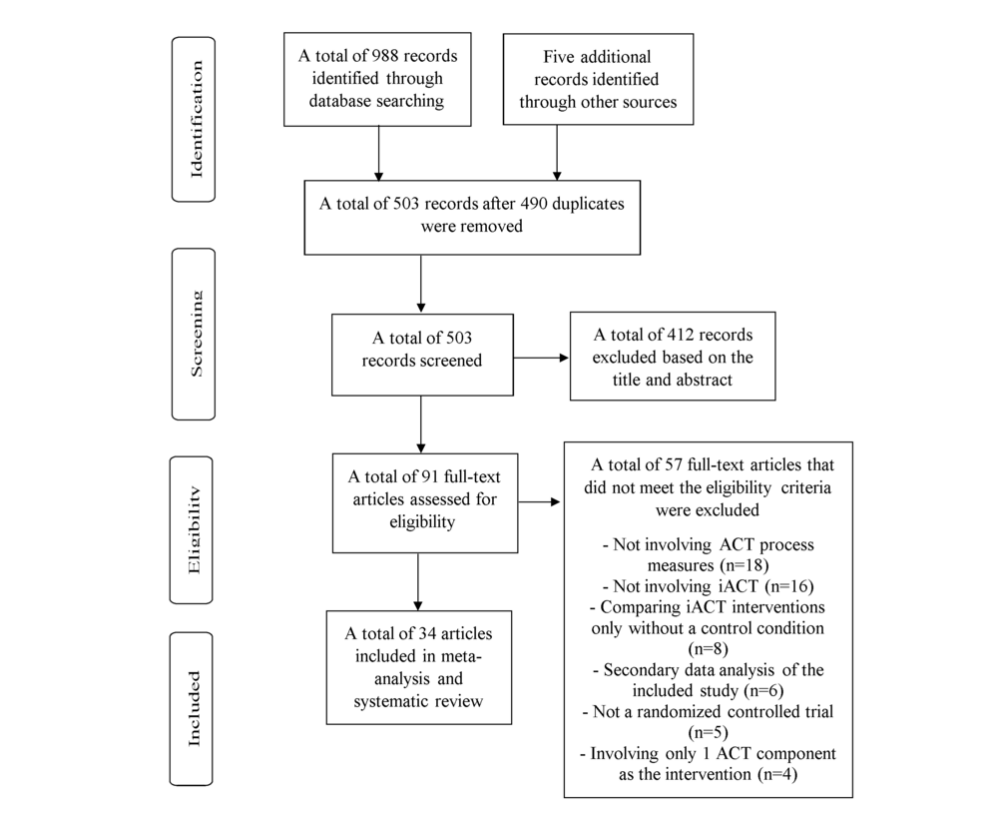
PRISMA (Preferred Reporting Items for Systematic Reviews and Meta-Analyses) flow diagram of the study selection process. ACT: acceptance and commitment therapy; iACT: internet-based acceptance and commitment therapy.

Figure 1 illustrates the study selection process. A total of 988 articles were identified through database searching, and 5 additional articles were identified through hand searching. After removing 490 duplicates, 503 articles were screened based on their titles and abstracts. A total of 412 articles were excluded based on title and abstract screening, and 91 articles were assessed for eligibility by reading the full text. A total of 57 articles were excluded after reading the full text because of the following reasons: not involving any ACT process measures (18 studies), not involving iACT (16 studies), comparing iACT interventions delivered differently without a control condition (8 studies), involving secondary data analysis (6 studies), not an RCT (5 studies), and involving only 1 ACT component for the intervention (4 studies). A total of 34 articles met the eligibility criteria [8,25-57].

### Characteristics of the Included Studies

The main characteristics of the included RCTs are summarized in Multimedia Appendix 2. The average number of ACT modules (sessions) in the included studies was 6.4 (SD 2.5), ranging from 2 to 12 modules in total. The average duration of ACT modules (sessions) delivered in the included studies was 7.9 (SD 3.2) weeks, ranging from 2 to 15 weeks in total. ACT was delivered on the web with therapist guidance in 26 studies (eg, via videoconferencing, phone calls, written feedback, and a mobile app) and without therapist guidance in 8 studies [25-32]. A total of 7 studies used a blended ACT program, which involved both iACT and in-person sessions [33-40]. A total of 3 studies involved videoconferencing ACT [41,42]. The remaining 24 RCTs used web-based ACT modules. Out of a total of 34 studies, 11 (32%) involved active control groups, including web-based cognitive behavioral therapy (CBT) [25], web-based smoking cessation interventions [26,27], web-based discussion forums [44,45,57], web-based mental health education [30], in-person behavioral support [35], web-based expressive writing [48,55], and in-person documentary discussion [38]. The population of the included studies varied widely, including college students [8,29-32,36,47,54], adults with chronic pain [37,39,52,55-57], family caregivers of people with chronic conditions [42,49,50,53], adults with insomnia [28,33,41], and smokers [26,27,35] (refer to Multimedia Appendix 2 for these different participant characteristics in the included studies). A total of 14 studies directly targeted people with certain types of psychological distress, such as depressive symptoms, anxiety, stress, and overall psychological distress [8,27,33,36-39,42,44-49].

The average sample size of participants in the included RCTs was 141 (SD 196), ranging from 24 to 1162. The mean age of the participants was 37.8 (SD 13.2) years, ranging from 13.9 to 55.9 years, and the average percentage of female participants was 68.1% (SD 19.7%), ranging from 0% to 98.5%. The included RCTs were conducted in the United States (10 studies), Finland (5 studies), Sweden (4 studies), the Netherlands (4 studies), the United Kingdom (2 studies), Australia (2 studies), Ireland (2 studies), Canada (1 study), Belgium (1 study), France (1 study), Denmark (1 study), and Germany (1 study). Of the 34 included studies, 26 (76%) were published between 2016 and 2021 and the remaining 8 (24%) were published between 2012 and 2015.

The following sections describe the results of the meta-analysis of the efficacy of iACT for psychological flexibility, mindfulness, valued living, and cognitive defusion at the immediate posttest and at follow-up, with findings of subgroup analyses listed according to the type of control group (ie, subgroup 1: iACT vs active control groups and subgroup 2: iACT vs passive control groups), when applicable.

### Effects of iACT on Improving Psychological Flexibility at the Immediate Posttest

**Figure 2:**
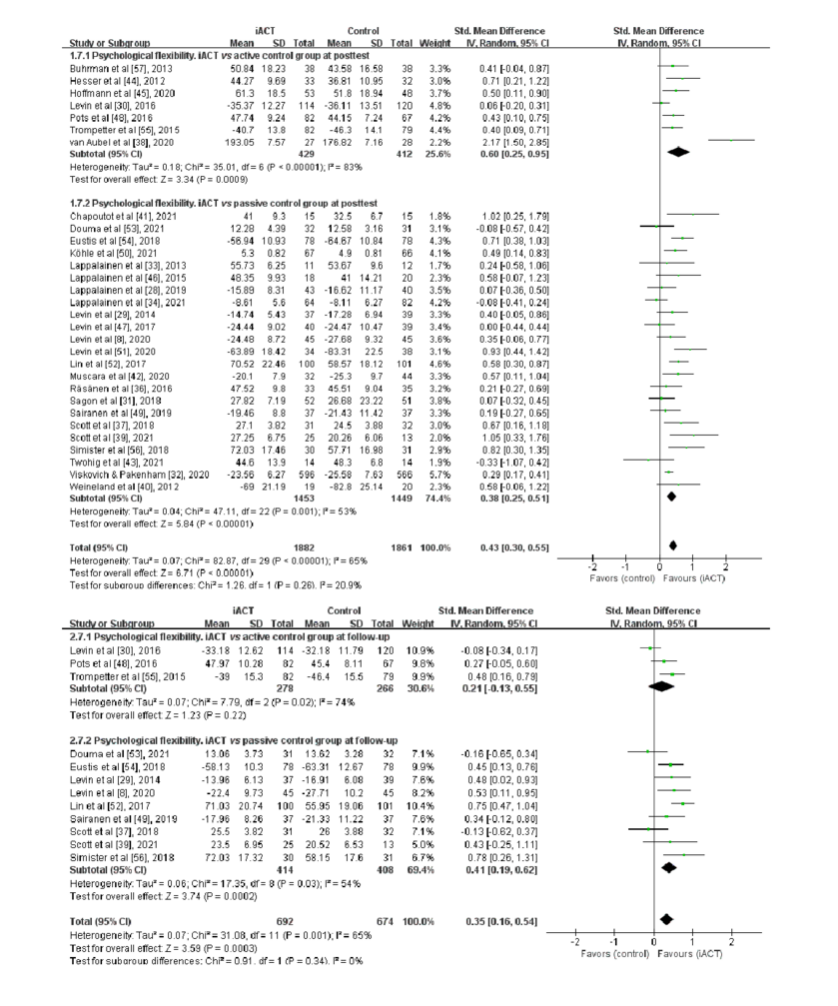
Forest plots showing the effects of internet-based acceptance and commitment therapy on psychological flexibility at the immediate posttest and at follow-up. iACT: internet-based acceptance and commitment therapy.

A meta-analysis of 30 RCTs (n=3743 participants) found that iACT had a medium effect on improving psychological flexibility at the immediate posttest compared with control groups overall (SMD=0.43, 95% CI 0.30-0.55; Figure 2). There was no significant subgroup difference at the immediate posttest (χ^2^_1_=1.26; *P*=.26), indicating that the effects of the 2 subgroups (ie, subgroup 1: iACT vs active control groups and subgroup 2: iACT vs passive control groups) at the immediate posttest were not statistically different from one another. The iACT had a medium effect on psychological flexibility compared with active control groups at the immediate posttest (7 studies that involved 841 participants; SMD=0.60, 95% CI 0.25-0.95), whereas iACT had a small effect compared with passive control groups (23 studies that involved 2902 participants; SMD=0.38, 95% CI 0.25-0.51).

### Effects of iACT on Improving Psychological Flexibility at Follow-up

A meta-analysis of 12 RCTs with follow-up data (n=1366 participants) revealed that iACT had a small effect on improving psychological flexibility at follow-up compared with control groups overall (SMD=0.35, 95% CI 0.16-0.54; Figure 2). There was no significant subgroup difference at follow-up (χ^2^_1_=0.91; *P*=.34), indicating that the effects of the 2 subgroups (ie, subgroup 1: iACT vs active control groups and subgroup 2: iACT vs passive control groups) at follow-up were not statistically different from one another. The iACT had a medium effect on improving psychological flexibility compared with passive control groups at follow-up (9 studies that involved 822 participants; SMD=0.41, 95% CI 0.19-0.62), but iACT was not significantly different from active control groups (3 studies that involved 544 participants; SMD=0.21, 95% CI −0.13 to 0.55).

### Effects of iACT on Improving Mindfulness at the Immediate Posttest

**Figure 3:**
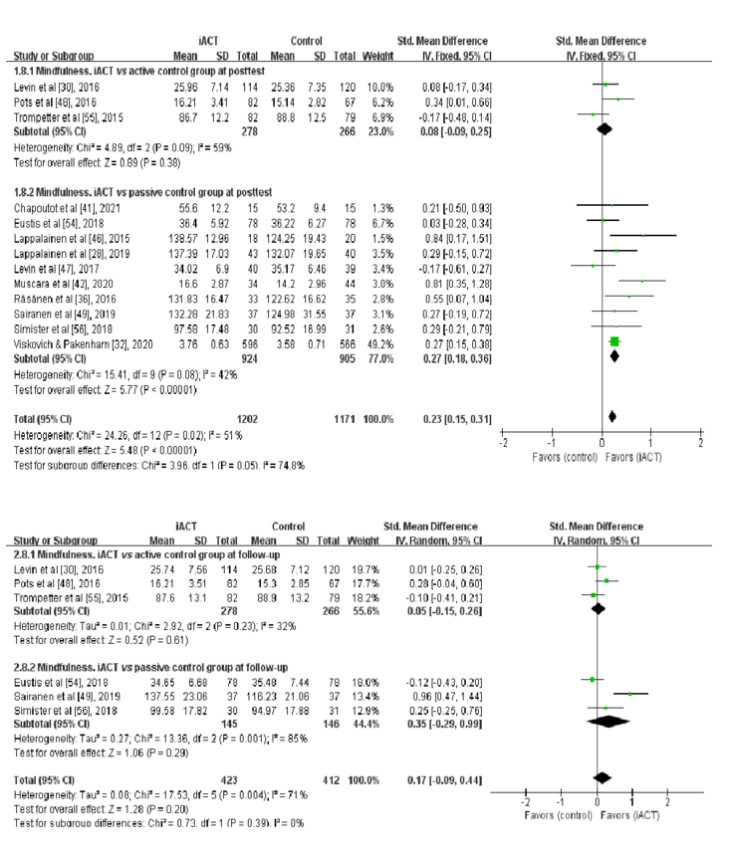
Forest plots showing the effects of internet-based acceptance and commitment therapy on mindfulness at the immediate posttest and at follow-up. iACT: internet-based acceptance and commitment therapy.

A meta-analysis of 13 RCTs (n=2373 participants) showed that iACT had a small effect on improving mindfulness at the immediate posttest compared with control groups overall (SMD=0.23, 95% CI 0.15-0.31; Figure 3). There was a significant subgroup difference at the immediate posttest (χ^2^_1_=2.96; *P*=.05), indicating that the effects of the 2 subgroups (ie, subgroup 1: iACT vs active control groups and subgroup 2: iACT vs passive control groups) at the immediate posttest were statistically different from one another. The iACT had a small effect on improving mindfulness compared with passive control groups at the immediate posttest (10 studies that involved 1829 participants; SMD=0.27, 95% CI 0.18-0.36), but iACT was not significantly different from active control groups (3 studies that involved 544 participants; SMD=0.08, 95% CI −0.09 to 0.25).

### Effects of iACT on Improving Mindfulness at Follow-up

A meta-analysis of 6 RCTs with follow-up data (n=835 participants) found that iACT was not significantly different overall from control groups in improving mindfulness at follow-up (SMD=0.17, 95% CI −0.09 to 0.44; Figure 3). There was no significant subgroup difference at follow-up (χ^2^_1_=0.73; *P*=.39), indicating that the effects of the 2 subgroups (ie, subgroup 1: iACT vs active control groups and subgroup 2: iACT vs passive control groups) at follow-up were not statistically different from one another. No significant between-group difference in improving mindfulness was found at follow-up, regardless of control group conditions, including 3 studies (n=544 participants) that compared iACT with active control groups (SMD=0.05, 95% CI −0.15 to 0.26) and 3 studies (n=291) that compared iACT with passive control conditions (SMD=0.35, 95% CI −0.29 to 0.99).

### Effects of iACT on Improving Valued Living at the Immediate Posttest

**Figure 4:**
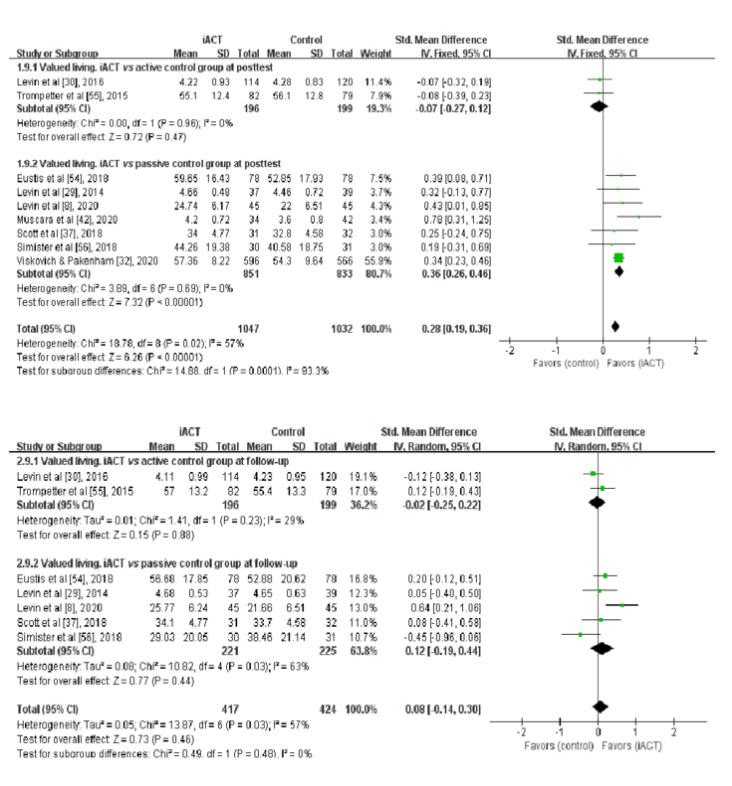
Forest plots showing the effects of internet-based acceptance and commitment therapy on valued living at the immediate posttest and at follow-up. iACT: internet-based acceptance and commitment therapy.

A meta-analysis of 9 RCTs (n=2079 participants) revealed that iACT had a small effect on improving valued living at the immediate posttest compared with control groups overall (SMD=0.28, 95% CI 0.19-0.36; Figure 4). There was a statistically significant subgroup difference at the immediate posttest (χ^2^_1_=14.88; *P*<.001), indicating that the effects of the 2 subgroups (ie, subgroup 1: iACT vs active control groups and subgroup 2: iACT vs passive control groups) at the immediate posttest were statistically different from one another. The iACT had a small effect on improving valued living compared with passive control groups at the immediate posttest (7 studies that involved 1684 participants; SMD=0.36, 95% CI 0.26-0.46), but iACT was not significantly different from active control groups (2 studies that involved 395 participants; SMD=−0.07, 95% CI −0.27 to 0.12).

### Effects of iACT on Improving Valued Living at Follow-up

A meta-analysis of 7 RCTs with follow-up data (n=841 participants) showed that iACT did not differ from control groups in improving valued living at follow-up (SMD=0.08, 95% CI −0.14 to 0.30; Figure 4). There was no statistically significant subgroup difference at follow-up (χ^2^_1_=0.49; *P*=.48), indicating that the effects of the 2 subgroups (ie, subgroup 1: iACT vs active control groups and subgroup 2: iACT vs passive control groups) at follow-up were not statistically different from one another. No significant between-group difference in improving valued living was found at follow-up, regardless of control group conditions, including 2 studies (n=395 participants) that compared iACT with active control groups (SMD=−0.02, 95% CI −0.25 to 0.22) and 5 studies (n=446 participants) that compared iACT with passive control conditions (SMD=0.12, 95% CI −0.19 to 0.44).

### Effects of iACT on Improving Cognitive Defusion at the Immediate Posttest

**Figure 5:**
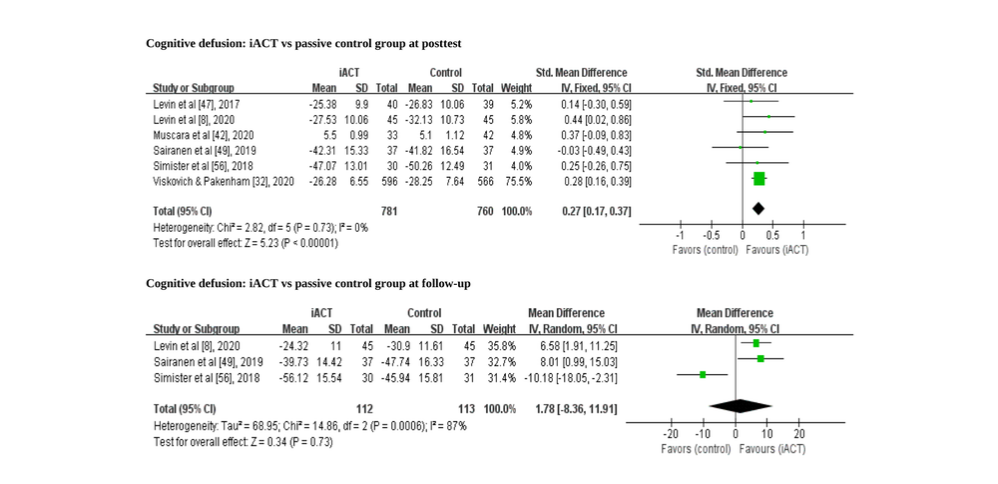
Forest plots showing the effects of internet-based acceptance and commitment therapy on cognitive defusion at the immediate posttest and at follow-up. iACT: internet-based acceptance and commitment therapy.

All studies that measured cognitive defusion compared iACT with passive control groups only; therefore, a subgroup analysis was not conducted. A meta-analysis of 6 RCTs (n=1541 participants) found that iACT had a small effect on improving cognitive defusion at the immediate posttest compared with passive control groups (SMD=0.27, 95% CI 0.17-0.37; Figure 5). A meta-analysis of 3 RCTs with follow-up data (n=225 participants) revealed that iACT was not different from passive control groups in improving cognitive defusion at follow-up (SMD=1.78, 95% CI −8.36 to 11.91; Figure 5).

### Subgroup Analyses According to the Use of Therapist Guidance

Subgroup analyses showed medium effects of iACT with therapist guidance on psychological flexibility at the immediate posttest (25 studies that involved 2085 participants; SMD=0.50, 95% CI 0.34-0.65) and at follow-up (10 studies that involved 1056 participants; SMD=0.40, 95% CI 0.21-0.58) and small effects of iACT with therapist guidance on mindfulness (10 studies that involved 894 participants; SMD=0.20, 95% CI 0.07-0.34) and valued living (7 studies that involved 686 participants; SMD=0.26, 95% CI 0.04-0.48) at the immediate posttest compared with control groups. The iACT with therapist guidance was not significantly different from the control groups in valued living at follow-up (5 studies that involved 531 participants, SMD=0.14, 95% CI −0.15 to 0.43). Conversely, subgroup analyses revealed small effects of iACT without therapist guidance on psychological flexibility (6 studies that involved 1700 participants, SMD=0.18, 95% CI 0.03-0.32) and mindfulness (3 studies that involved 1479 participants, SMD=0.24, 95% CI 0.14-0.34) at the immediate posttest only. The iACT without therapist guidance was not significantly different from control groups in psychological flexibility at follow-up (2 studies that involved 310 participants; SMD=0.17, 95% CI −0.38 to 0.71) and valued living at the immediate posttest (3 studies that involved 1472 participants; SMD=0.20, 95% CI −0.10 to 0.49) and follow-up (2 studies that involved 310 participants; SMD=−0.08, 95% CI −0.30 to 0.14). In particular, the pooled effect size of studies that involved iACT with therapist guidance was greater than that of studies that involved iACT without therapist guidance in psychological flexibility at the immediate posttest. In addition, although there was a statistically significant pooled effect of iACT with therapist guidance on valued living at the immediate posttest, iACT without therapist guidance was not significantly different from the control groups. There was no statistically significant subgroup difference in any of the outcomes (*P*>.05), except for psychological flexibility at the immediate posttest (*P*=.003). These findings indicate that there was no statistically significant difference among studies according to the use of therapist guidance in all outcomes, except for psychological flexibility at the immediate posttest, in which a statistically significant larger effect of iACT was found when iACT studies involved therapist guidance (ie, SMD=0.50 vs SMD=0.18). Forest plots of these subgroup analyses are illustrated in Multimedia Appendices 3-7.

### Subgroup Analyses According to the Delivery Modes

Subgroup analyses showed small effects of web-based ACT on psychological flexibility (22 studies that involved 3257 participants; SMD=0.38, 95% CI 0.26-0.49) and mindfulness at the immediate posttest (10 studies that involved 2197 participants; SMD=0.20, 95% CI 0.11-0.28) compared with control groups. Subgroup analyses revealed medium effects of iACT accompanied by in-person ACT sessions on psychological flexibility (6 studies that involved 394 participants; SMD=0.61, 95% CI 0.02-1.20) and mindfulness (1 study that involved 68 participants; SMD=0.55, 95% CI 0.07-1.04) at the immediate posttest compared with control groups. Although videoconferencing ACT was not significantly different from control groups in psychological flexibility at the immediate posttest (3 studies that involved 134 participants; SMD=0.43, 95% CI −0.25 to 1.11), subgroup analyses found medium effects of videoconferencing ACT on mindfulness at the immediate posttest compared with control groups (2 studies that involved 108 participants; SMD=0.64, 95% CI 0.24-1.03). There was no statistically significant subgroup difference in psychological flexibility at the immediate posttest (*P*>.05), indicating that there was no statistically significant difference among studies according to delivery modes. However, there was a statistically significant subgroup difference in mindfulness at the immediate posttest (*P*=.04), suggesting a statistically significant difference among studies according to their delivery modes, in which medium effects of iACT with in-person sessions and videoconferencing ACT were found, whereas web-based ACT showed small effects. Subgroup analyses according to the delivery modes were not conducted for the other outcomes because of the lack of studies on any other outcomes. Forest plots of these subgroup analyses are illustrated in Multimedia Appendices 8 and 9.

### Subgroup Analyses According to the Targeted Participants With Psychological Distress Symptoms

Subgroup analyses found a medium effect of iACT on psychological flexibility at the immediate posttest (13 studies that involved 919 participants; SMD=0.55, 95% CI 0.31-0.79) and small effects of iACT on psychological flexibility at follow-up (5 studies that involved 414 participants; SMD=0.29, 95% CI 0.09-0.49) and on mindfulness (6 studies that involved 486 participants; SMD=0.38, 95% CI 0.20-0.56), valued living (4 studies that involved 308 participants; SMD=0.35, 95% CI 0.02-0.69), and cognitive defusion (4 studies that involved 318 participants; SMD=0.24, 95% CI 0.02-0.46) at the immediate posttest compared with control groups when studies directly targeted participants with some type of psychological distress. Subgroup analyses, however, showed small effects of iACT on psychological flexibility at the immediate posttest (18 studies that involved 2866 participants; SMD=0.33, 95% CI 0.19-0.48) and at follow-up (7 studies that involved 952 participants; SMD=0.38, 95% CI 0.10-0.67), mindfulness at the immediate posttest (7 studies that involved 1887 participants; SMD=0.19, 95% CI 0.10-0.28), and cognitive defusion at the immediate posttest (2 studies that involved 1223 participants; SMD=0.28, 95% CI 0.16-0.39) compared with control groups in studies that did not involve targeted participants with some type of psychological distress. However, there was no statistically significant difference in iACT among control groups in mindfulness and valued living at follow-up when studies did or did not directly target participants with some type of psychological distress, or in valued living at the immediate posttest when studies did not involve targeted participants with some type of psychological distress. There were no statistically significant subgroup differences in any of the outcomes (*P*>.05), indicating that there was no statistically significant difference among studies according to the use of targeted participants with some type of psychological distress in all the outcomes. Forest plots of these subgroup analyses are illustrated in Multimedia Appendices 10-16.

### Risk of Bias and Publications Bias of the Included Studies

Out of the 34 included studies, 18 (53%) had an unclear risk of bias, 10 (29%) had a low risk of bias, and 6 (18%) had a high overall risk of bias (Multimedia Appendix 17). A domain regarding blinding of participants and personnel was not regarded as the key domain for the overall risk of bias within a study because studies that involved passive control conditions were less able to conceal the group allocation from participants. The overall risk of bias across the 34 studies was interpreted as unclear because most information was from studies with an unclear risk of bias [24]. The risk of bias in each domain for each study is reported in Multimedia Appendix 17, and the risk of bias summary graph and chart are reported in Multimedia Appendix 18 [8,25-57] and Multimedia Appendix 19.

As at least 10 studies have suggested using the funnel plot asymmetry analysis, possible publication bias was tested for psychological flexibility and mindfulness at posttest only [24]. Overall, the funnel plots tend to be symmetrical, although more studies are needed to better interpret such visual inspection, especially for mindfulness. Funnel plots are reported in Multimedia Appendix 20.

## Discussion

This systematic review and meta-analysis identified 34 RCTs that assessed the efficacy of iACT for process measures (ie, psychological flexibility, mindfulness, valued living, and cognitive defusion). This meta-analysis found that iACT had a medium effect on improving psychological flexibility and small effects on improving mindfulness, valued living, and cognitive defusion at the immediate posttest. A small effect of iACT on psychological flexibility was also observed at follow-up.

Previous meta-analysis studies that involved ACT (ie, not iACT in particular) found similar findings to this meta-analysis study that included a medium effect of ACT on psychological flexibility in family caregivers [6] and a small effect of self-help ACT on psychological flexibility in adult populations [58]. A total of 26 studies that assessed the effects of iACT on process measures were conducted from 2016 to the end date of the search for this meta-analysis (June 5, 2021), whereas only 8 studies were published before 2016. This may explain why a previous meta-analysis study of iACT in particular did not include process measures [21]. Thompson et al [22] conducted a meta-analysis for psychological flexibility only among process measures and found small effects of iACT on psychological flexibility at the immediate posttest and at follow-up. These findings by Thompson et al [22] were based on 23 studies at immediate posttest and 11 studies at follow-up. This meta-analysis found a medium effect at the immediate posttest (based on 30 studies) and a small effect at follow-up (based on 12 studies). The larger effect size of iACT on psychological flexibility at the immediate posttest found in this study may be because of the inclusion of 7 more studies than the meta-analysis by Thompson et al [22]. Unlike previous meta-analysis studies for iACT [21,22], this meta-analysis study conducted meta-analyses in other process measures, such as mindfulness, valued living, and cognitive defusion, and found small effects on these measures at the immediate posttest. Thus, the findings of this meta-analysis contribute to the body of evidence on the efficacy of iACT for different process measures, which could be applicable to both clinical and nonclinical populations, as ACT is a transdiagnostic approach [1].

Subgroup analyses for each outcome were conducted in this meta-analysis study according to the type of control group, unlike previous iACT meta-analysis studies [21,22]. Subgroup analyses showed small effects of iACT on psychological flexibility, mindfulness, valued living, and cognitive defusion at the immediate posttest compared with passive control groups. In contrast, subgroup analyses that compared iACT with active control groups found no significant between-group differences in mindfulness and valued living at the immediate posttest and follow-up and in psychological flexibility at follow-up. Such findings indicate that iACT was not significantly more effective than active control conditions (eg, CBT and mental health education). However, relatively few studies have compared the effects of iACT with active control conditions. There were 3 times more studies that compared iACT with passive control conditions than those that compared iACT with active control conditions in all the process measures, except for mindfulness at follow-up. Such a gap in the literature suggests a need for studies that compare iACT with active control conditions, such as CBT, psychoeducational interventions, and support groups, to better understand whether iACT is comparable or superior to other evidence-based treatments in improving process measures such as mindfulness and cognitive defusion.

This study conducted subgroup analyses according to the use of therapist guidance, delivery modes, and targeted participants with symptoms of psychological distress. This study found no statistically significant subgroup difference among studies of these 3 characteristics in all the outcomes, except for the subgroup difference among studies according to the use of therapist guidance for psychological flexibility at the immediate posttest (ie, a larger effect of iACT when iACT was provided with therapist guidance compared with iACT without therapist guidance) and for the subgroup difference among studies according to the delivery modes for mindfulness at the immediate posttest (ie, a larger effect of iACT when iACT was delivered with in-person sessions or videoconferencing ACT compared with web-based ACT modules). Thompson et al [22] also found larger effects of iACT with therapist guidance on psychological flexibility compared with iACT without therapist guidance. However, more studies are needed to confirm these findings, especially in subgroup analyses according to delivery modes, because only a few studies have involved delivery modes other than web-based ACT modules.

This review had several limitations that should be considered when interpreting the findings. A total of 4 electronic databases were used to search the literature, and some relevant articles could have been missed if they were published only in other databases. Only studies written in English were searched and included in this review, which could create a publication bias. One author with extensive experience in comprehensive literature reviews and expertise in ACT searched the literature; therefore, this review did not include 2 independent reviewers in the search process. A recent systematic review found that single screening for study selection in systematic reviews conducted by experienced reviewers had no impact on the findings of the meta-analysis [59]. The overall risk of bias across the included RCTs was interpreted as unclear, indicating the need for high-quality studies to better determine the effects of iACT on process measures.

According to this meta-analysis study, there were 2 to 5 times more studies that assessed psychological flexibility than studies that assessed mindfulness, valued living, and cognitive defusion. Investigators of further studies should consider assessing diverse ACT process measures to better understand the processes of change. In addition, relatively few studies have been conducted to compare the effects of iACT with active control groups. Future high-quality studies that compare iACT with active control conditions are needed to better understand whether iACT is comparable or superior to other evidence-based treatments in process measures. The findings of this review contribute to the literature by showing the direct effects of iACT on ACT processes, which are theorized to foster improvements in mental health outcomes. These synthesized findings support the processes of change in iACT, which mental health practitioners can use to support the use of iACT.

## Appendix

Multimedia Appendix 1 Search terms used in database searches.

Multimedia Appendix 2 Characteristics of the included studies.

Multimedia Appendix 3 Forest plots showing effects of internet-based acceptance and commitment therapy on psychological flexibility according to the use of therapist guidance at the immediate posttest.

Multimedia Appendix 4 Forest plots showing effects of internet-based acceptance and commitment therapy on psychological flexibility according to the use of therapist guidance at follow-up.

Multimedia Appendix 5 Forest plots showing effects of internet-based acceptance and commitment therapy on mindfulness according to the use of therapist guidance at the immediate posttest.

Multimedia Appendix 6 Forest plots showing effects of internet-based acceptance and commitment therapy on valued living according to the use of therapist guidance at the immediate posttest.

Multimedia Appendix 7 Forest plots showing effects of internet-based acceptance and commitment therapy on valued living according to the use of therapist guidance at follow-up.

Multimedia Appendix 8 Forest plots showing effects of internet-based acceptance and commitment therapy on psychological flexibility according to delivery modes at the immediate posttest.

Multimedia Appendix 9 Forest plots showing effects of internet-based acceptance and commitment therapy on mindfulness according to delivery modes at the immediate posttest.

Multimedia Appendix 10 Forest plots showing effects of internet-based acceptance and commitment therapy on psychological flexibility according to the use of targeted distressed participants at the immediate posttest.

Multimedia Appendix 11 Forest plots showing effects of internet-based acceptance and commitment therapy on psychological flexibility according to the use of targeted distressed participants at follow-up.

Multimedia Appendix 12 Forest plots showing effects of internet-based acceptance and commitment therapy on mindfulness according to the use of targeted distressed participants at the immediate posttest.

Multimedia Appendix 13 Forest plots showing effects of internet-based acceptance and commitment therapy on mindfulness according to the use of targeted distressed participants at follow-up.

Multimedia Appendix 14 Forest plots showing effects of internet-based acceptance and commitment therapy on valued living according to the use of targeted distressed participants at the immediate posttest.

Multimedia Appendix 15 Forest plots showing effects of internet-based acceptance and commitment therapy on valued living according to the use of targeted distressed participants at follow-up.

Multimedia Appendix 16 Forest plots showing effects of internet-based acceptance and commitment therapy on cognitive defusion according to the use of targeted distressed participants at the immediate posttest.

Multimedia Appendix 17 Risk of bias of the included studies.

Multimedia Appendix 18 Risk of bias summary chart.

Multimedia Appendix 19 Risk of bias graph.

Multimedia Appendix 20 Funnel plot: Psychological flexibility and Mindfulness.

## Abbreviations

ACT: acceptance and commitment therapy
CBT: cognitive behavioral therapy
iACT: internet-based acceptance and commitment therapy
PRISMA: Preferred Reporting Items for Systematic Reviews and Meta-Analyses
RCT: randomized controlled trial
SMD: standardized mean difference
