# Supplementary material for: The Effects of Internet-Based Acceptance and Commitment Therapy on Process Measures: Systematic Review and Meta-analysis
Source: J Med Internet Res. 2022 Aug 30;24(8):e39182. doi: 10.2196/39182 (PMC9472046; doi:10.2196/39182)
Supplement: Multimedia Appendix 1 [file jmir_v24i8e39182_app1.docx]

**Search Terms Used in Database Searches**

| Database | Search terms |
| --- | --- |
| PubMed | (“acceptance and commitment therapy”[tiab] OR “Acceptance and Commitment Therapy”[MeSH]) AND (online[tiab] OR e-health[tiab] OR Internet*[tiab] OR web[tiab] OR webs[tiab] OR “web-based”[tiab] OR “web-delivered”[tiab] OR computer*[tiab] OR app[tiab] OR apps[tiab] OR mobile[tiab] OR technolog*[tiab] OR “Computers”[Mesh] OR “Internet-Based Intervention”[MeSH] OR “Telemedicine”[MeSH] OR “Distance Counseling”[MeSH] OR “Mobile Applications”[Mesh]) |
| CINAHL | (TI "acceptance and commitment therapy" OR AB "acceptance and commitment therapy") AND (TI "online" OR AB "online" OR TI "e-health" OR AB "e-health" OR TI "internet*" OR AB "internet*" OR TI "web*" OR AB "web*" OR TI "computer*" OR AB "computer*" OR TI "app" OR AB "app" OR TI "apps" OR AB "apps" OR TI "mobile" OR AB "mobile" OR TI "technolog*" OR AB "technolog*") |
| PsycInfo® | (TIAB("acceptance and commitment therapy")) AND (TIAB(online OR e-health OR Internet* OR web* OR computer* OR app OR apps OR mobile OR technolog*)) |
| Scopus | ( TITLE-ABS-KEY ( "acceptance and commitment therapy" )  AND  TITLE-ABS-KEY ( online  OR  e-health OR internet* OR web* OR computer* OR app OR apps OR mobile OR technolog*) ) |
