# Supplementary material for: The Effects of Internet-Based Acceptance and Commitment Therapy on Process Measures: Systematic Review and Meta-analysis
Source: J Med Internet Res. 2022 Aug 30;24(8):e39182. doi: 10.2196/39182 (PMC9472046; doi:10.2196/39182)
Supplement: Multimedia Appendix 2 [file jmir_v24i8e39182_app2.docx]

**Characteristics of the Included Studies**

| Reference; Country | Participants; Mean age; Female (%) | Descriptions of intervention and control groups | Relevant outcomes (measures); Data collection time points; Relevant results (between-group differences only) | Overall RoB |
| --- | --- | --- | --- | --- |
| Barrett & Stewart (2021); Ireland | 42 adults working within the social and healthcare professions; 37.1 years; 88% | - IG: A 2-week online ACT program (3 sessions) for stress management, involving videos, brief informational questions that tracked understanding of and attention to the material, homework exercises, and ACT-based mindfulness exercises (*n* = 22)  - CG: A 2-week online CBT intervention for stress management, involving 3 sessions (*n* = 20) | Work-related psychological flexibility/acceptance (WAAQ); Pretest and posttest; No significant between-group differences on WAAQ over time | Unclear |
|  |  |  |  |  |
| Bricker et al. (2013); USA | 222 adults who smoke at least 5 cigarettes daily for at least the past 12 months; 45.1 years; 62% | - IG: An 8-part, self-paced, web-based ACT program for smoking cessation (WebQuit) for 3 months (*n* = 111)  - CG: National Cancer Institute's Smokefree.gov, the US national standard for web-based smoking cessation interventions covering quit planning, skills training, advice on pharmacotherapy, and social support for quitting (*n* = 111) | Psychological flexibility/acceptance (AIS-27); Pretest and posttest; A nonsignificant trend of greater acceptance in WebQuit.org (IG)  quitters than in the Smokefree.gov (CG) quitters | High |
|  |  |  |  |  |
| Buhrman et al. (2013); Sweden | 76 adults with functional impairments caused by chronic pain; 49.1 years; 59.2% | - IG: A 7-week guided internet-delivered ACT intervention for chronic pain, involving information about the ACT processes, assignments, relevant metaphors, and mindfulness exercises that could be downloaded as MP3 files + a text message as a reminder for homework assignments + 30-minute phone calls at weeks 3 and 7 (*n* = 38)  - CG: A moderated online discussion forum for chronic pain presenting weekly discussion topics by therapists and encouraging discussion (*n* = 38) | Psychological flexibility/acceptance (CPAQ); Pretest, posttest, and 6-month F/U; A significant improvement on CPAQ in IG compared to CG over time (*p* < 0.05) | Low |
|  |  |  |  |  |
| Chapoutot et al. (2021); France | 32 adults with chronic insomnia and hypnotic dependence; 48 years; 80% | - IG: Four 1-hour individual videoconference sessions delivered every 2 weeks by 2 psychologists trained in ACT and CBT for insomnia treatment + an ACT manual and audio recordings of mindfulness exercises (*n* = 16)  - CG: Waitlist control (*n* = 16) | Psychological flexibility/ acceptance (AAQ-II) and mindfulness (MAAS); Pretest, posttest, and 6-month F/U; A significant improvement on AAQ-II in IG compared to CG over time (*p* < 0.001) | Unclear |
|  |  |  |  |  |
| Douma et al. (2021); the Netherlands | 73 parent caregivers of children with a physical chronic illness; 42.5 years; 98.5% | - IG: 6 weekly 90-min group sessions based on ACT and CBT delivered in a secured chatroom with 3 to 5 parents guided by 2 psychologists + a booster session at 4 months after the last regular session aiming to prevent and/or reduce psychosocial problems by teaching the use of adaptive disease-related coping skills (*n* = 39)  - CG: Waitlist control (*n* = 34) | Acceptance (Acceptance subscale of the ICQ-P); Baseline, 6-month F/U, and 12-month F/U; A significant improvement on ICQ-P-acceptance in IG compared to CG at 6-month F/U (*p* < 0.05) | High |
|  |  |  |  |  |
| Eustis et al. (2018); USA | 156 college students; 25.4 years; 78.8% | - IG: A 3-session web-based therapist-assisted ACT targeting anxiety, focusing on psychoeducation, mindfulness, and values, in which each session includes two 15-min narrated PowerPoint slides with text, images, and experiential exercises and a written practice assignment about related skill practice and/or barriers to skill practice + an email reminder + written feedback and support from the therapist (*n* = 78)  - CG: Waitlist control (*n* = 78) | Psychological flexibility/ acceptance (AAQ), mindfulness (PHLMS), and valued living (VLQ); Pretest, posttest, and 1-month F/U; Significant improvements on AAQ (*p* < 0.001) and VLQ (*p* < 0.05) in IG compared to CG over time | High |
|  |  |  |  |  |
| Heffner et al. (2020); USA | 51 daily smokers with bipolar I or II disorder; 49 years; 45% | - IG: an 8-part, self-paced, web-based ACT program for smoking cessation involving ACT exercises and psychoeducation to address specific challenges to smoking cessation for smokers with bipolar disorders (WebQuit Plus) over a 10-week treatment period + weekly email reminders containing a link to the assigned program and daily text messages for 70 days with links to the program + nicotine patch for 8 weeks (*n* = 25)  - CG: National Cancer Institute's Smokefree.gov, the US national standard for web-based smoking cessation interventions covering quit planning, skills training, advice on pharmacotherapy, and social support for quitting over a 10-week treatment period + weekly email reminders containing a link to the assigned program and daily text messages for 70 days with links to the program + nicotine patch for 8 weeks (*n* = 26 ) | Psychological flexibility/acceptance (AIS-27); Committed action (CQS); Pretest, posttest, and 1-month F/U; No significant between-group differences on AIS-27 and CQS over time | High |
|  |  |  |  |  |
| Hesser et al. (2012); Sweden | 99 adults with moderate to severe distress due to tinnitus; 48.5 years; 43.4% | - IG1: 8 weekly modules of guided internet-delivered ACT involving structured self-help material presented via the internet, an identified therapist who provided support and guidance, mindfulness and defusion exercises that could be downloaded as MP3 files, homework assignments, and email reminders (*n* = 35)  - IG2: 8 weekly modules of guided internet-delivered CBT in guided self-help format including tinnitus-specific CBT techniques (eg, applied relaxation, positive imagery, attention training, cognitive restructuring), homework assignments, and email reminders (*n* = 32)  - CG: A moderated online discussion forum that specifically targeted tinnitus-related problems, presented weekly discussion topics by therapists, and encouraged discussion (*n* = 32) | Acceptance in relation to tinnitus (TAQ); Pretest, posttest, and 12-month F/U; A significant improvement on TAQ in IG1 compared to CG over time (*p* < 0.05) | Unclear |
|  |  |  |  |  |
| Hoffmann et al. (2020); Denmark | 101 adults experiencing severe health anxiety (hypochondriasis); 39.8 years; 65.3% | - IG: 7 modules of a clinician-guided, self-help internet-delivered ACT program over 12 weeks, developed as a web app, involving text, illustrations, audio files (mindfulness exercises), video clips, interactive worksheets, and an encrypted and embedded message system enabling written communication (*n* = 53)  - CG: 7 internet-delivered discussion forums available over 12 weeks with a new topic each week related to health anxiety, such as health care, relationships, or work (*n* = 48) | Psychological flexibility/ acceptance (AAQ-II); Pretest, posttest, and 6-month F/U; A significant improvement on AAQ-II in IG compared to CG over time (*p* < 0.001) | Low |
|  |  |  |  |  |
| Köhle et al. (2021); the Netherlands | 203 partners of cancer patients; 55.9 years; 70.4% | - IG1: 6 modules (+2 optional modules) of web-based self-help ACT over 12 weeks, with personal feedback on participants' experiences with the module, progress in the module, and feedback on key eservices via weekly email messages from a personal counselor (*n* = 67)  - IG2: 6 modules of web-based self-help ACT over 12 weeks, with short, preprogrammed feedback messages aiming to normalize and validate emotions and reactions participants could experience after completing key exercises of the module (*n* = 70)  - CG: Waitlist control (*n* = 66) | Psychological flexibility/acceptance (AAQ-II); Pretest, posttest, and 3-month F/U; A significant improvement on AAQ-II in IG1 compared to CG over time (*p* < 0.05) | Low |
|  |  |  |  |  |
| Lappalainen et al. (2013); Finland | 24 working-age males experiencing exhaustion, stress symptoms, or sleeping problems; 43.3 years; 0% | - IG: An ACT-based program that integrated different personal health technologies, including a web portal, mobile phone applications, personal monitoring devices, and analysis software, with three 2-hour group meetings held by a psychologist (*n* = 12)  - CG: Waitlist control (*n* = 12) | Psychological flexibility/ acceptance (AAQ-II); Pretest, posttest, and 6-month F/U; No significant between-group differences on AAQ-II over time | Unclear |
|  |  |  |  |  |
| Lappalainen et al. (2015); Finland | 39 adults with major depressive episode; 51.9 years; 71.8% | - IG: 6 weekly web-based ACT modules, involving self-help texts, videos, and downloadable MP3 audio files for mindfulness and other experiential exercises, home assignments, online personalized written feedback from trained master's-level students of psychology, and automated email-based reminders over a 7-week intervention period (*n* = 19)  - CG: Waitlist control (*n* = 20) | Psychological flexibility/ acceptance (AAQ-II) and mindfulness (FFMQ); Pretest, posttest, and 12-month F/U; Significant improvements on AAQ-II (*p* < 0.01) and FFMQ (*p* < 0.05) in IG compared to CG over time | Unclear |
|  |  |  |  |  |
| Lappalainen et al. (2019); Finland | 83 adults with clinical insomnia; 53.5 years; 63.9% | - IG: 6 weekly self-help web-based ACT modules for sleep disturbances with 2 email-based automated reminders were sent every week, involving text, experiential audio exercises, and video clips (*n* = 43)  - CG: Waitlist control (*n* = 40) | Psychological flexibility/acceptance (AAQ-II) and mindfulness (FFMQ); Pretest, posttest, and 6-month F/U; No significant between-group differences on AAQ-II and FFMQ over time | Unclear |
|  |  |  |  |  |
| Lappalainen et al. (2021); Finland | 243 adolescents aged 15-16 years; 15.3 years; 51% | - IG1: 5 weekly modules of a web-based ACT intervention (Youth COMPASS), involving short texts, pictures, video clips, comic strips, audio-based exercises, and homework assignments, with face-to-face support (two 45-minute individual face-to-face meetings) and brief weekly feedback provided by a trained coach via WhatsApp (*n* = 81)  - IG2: a 5-week web-based ACT intervention (Youth COMPASS) with brief weekly feedback provided by an individual coach only (no face-to-face sessions) via WhatsApp (*n* = 80)  - CG: Usual care (*n* = 82) | Psychological flexibility/acceptance (AFQ-Y); Pretest and posttest; No significant between-group differences on AFQ-Y over time | Unclear |
|  |  |  |  |  |
| Levin et al. (2014); USA | 76 undergraduate first-year students; 18.4 years; 53.9% | - IG: web-based ACT, involving 2 web-based lessons, such as animations, audio narration, text and graphic elements, interactive metaphors, experiential exercises, and interactive assessments and supplementary tailored emails over a 3-week intervention period (*n* = 37)  - CG: Waitlist control (*n* = 39) | Psychological flexibility/acceptance (AAQ-II) and valued living (PVQ); Pretest, posttest, and 3-week F/U; No significant between-group differences on AAQ-II and PVQ over time | Unclear |
|  |  |  |  |  |
| Levin et al. (2016); USA | 234 college students; 21.6 years; 76.9% | - IG: A 3-week web-based ACT program focusing on acceptance and values, involving 2 core multimedia sessions (audio narration, animation, text and graphic elements, and interactive exercises), supplementary emails, web-based resources, and text messages with reminder emails and phone calls (*n* = 114)  - CG: A 3-week web-based mental health education program, involving 2 web-based sessions that focused on providing basic educational information about the symptoms and causes of depression and anxiety and brief information on coping strategies (*n* = 120) | Psychological flexibility/acceptance (AFQ-Y), mindfulness (FFMQ), and valued living (PVQ); Pretest, posttest, 1-month F/U, and 3-month F/U; No significant between-group differences on AFQ-Y, FFMQ, and PVQ over time | Unclear |
|  |  |  |  |  |
| Levin et al. (2017); USA | 79 college students experiencing psychological distress; 20.5 years; 66% | - IG: 6 sessions of a web-based self-help ACT, involving text, images, audio recordings, and videos for experiential  exercises, worksheets, assessments with tailored feedback and expandable text/popup features, over a 4-week intervention period with reminder prompts via email and phone by research assistants (*n* = 40)  - CG: Waitlist control (*n* = 39) | Psychological flexibility/acceptance (AAQ-II), mindfulness (PHLMS awareness subscale), cognitive fusion (CFQ), and valued living (VQ progress subscale); Pretest and posttest; No significant between-group differences on the outcomes over time | Unclear |
|  |  |  |  |  |
| Levin et al. (2020a); USA | 181 college students with clinically significant psychological distress; 22.3 years; 72.4% | - IG1: 12 self-guided web-based ACT sessions over 6 weeks, taking about 15-30 minutes per session, involving text, videos, audio-guided mindfulness exercises, worksheets with responsive feedback, ACT metaphors, other ACT skill practice exercises (eg, defusion exercises), and homework assignments, with regular email prompts to support engagement and 10-minute phone coaching per week (*n* = 45)  - IG2: 12 self-guided web-based ACT sessions, focusing on acceptance and defusion components of ACT, over 6 weeks (*n* = 45)  - IG3: 12 self-guided web-based ACT sessions, focusing on values and committed action components of ACT, over 6 weeks (*n* = 46)  - CG: Waitlist control (*n* = 45) | Psychological flexibility/acceptance (AAQ-II), valued living (VQ), and cognitive fusion (CFQ); Pretest, posttest, and 1-month F/U; Significant improvements on AAQ-II (*p* < 0.01), VQ (*p* < 0.001), and CFQ (*p* < 0.001) in IG1 compared to CG over time | Unclear |
|  |  |  |  |  |
| Levin et al. (2020b); USA | 79 adults who were overweight/obese; 39.6 years; 82.3% | - IG: 8 weekly modules of an online guided self-help ACT program, integrated with nutrition education and strategies to increase physical activity, involving text, videos, and interactive exercises, with weekly 10-minute phone coaching calls for support (*n* = 39)  - CG: Waitlist control (*n* = 40) | Psychological flexibility/acceptance for weight (AAQ-W); Pretest, posttest, and 2-month F/U; A significant improvement on AAQ-W in IG compared to CG over time (*p* < 0.001) | High |
|  |  |  |  |  |
| Lin et al. (2017); Germany | 302 adults with chronic pain and pain interference; 51.7 years; 84.1% | - IG1: 8 weekly modules of internet- and mobile-based ACT with personalized and standardized feedback via e-mail within 2 working days after completion of each module by e-coaches (psychologists) (*n* = 100)  - IG2: 8 weekly modules of internet- and mobile-based ACT without therapist guidance (*n* = 101)  - CG: Waitlist control (*n* = 101) | Pain acceptance (CPAQ); Pretest, posttest, and 4-month F/U; A significant improvement on CPAQ in IG1 compared to CG over time (*p* < 0.01) | Low |
|  |  |  |  |  |
| Muscara et al. (2020); Australia | 81 parents of children with a recently diagnosed life-threatening illness or injury, reporting elevated acute stress symptoms; 37.2 years; 80.2% | - IG: A 6-session ACT-based group intervention delivered via online videoconferences over the course of 8 weeks, involving five 90-minute consecutive weekly sessions, with a sixth and final session held 3 weeks after the fifth session, delivered by 2 trained mental health clinicians and provided with a session booklet and guided mindfulness CD and MP3 files (*n* = 37)  - CG: Waitlist control (*n* = 44) | Psychological flexibility/acceptance (AAQ-II), mindfulness (FFMQ-short form), and valued living (VQ); Pretest and posttest; Significant improvements on AAQ-II (*p* < 0.05), Nonjudging of inner experience subscale of the FFMQ-short form (*p* < 0.01), and VQ (*p* < 0.01) in IG compared to CG over time | High |
|  |  |  |  |  |
| O'Connor et al. (2020); Ireland | 150 adults smoking 10 or more cigarettes per day; 36 years; 52.7% | - IG1: Use of an ACT smartphone application with ACT exercises in audio or video format and accompanied by a text transcript in augmenting 6 weekly 1.5-hour in-person group sessions of ACT (*n* = 50)  -IG2: 6 weekly 1.5-hour in-person group sessions of ACT (*n* = 50)  - CG: 6 weekly 1.5-hour in-person group sessions of behavioral support delivered using core skills of motivational interviewing (*n* = 50) | Psychological flexibility/acceptance (AIS), mindfulness (Awareness subscale of the PHLMS), cognitive fusion (CFQ), and valued living (VQ); Pretest, posttest, and 6-month F/U; Significant improvements on AIS and Awareness subscale of the PHLMS in IG1 compared to CG over time (*p* < 0.05) | Low |
|  |  |  |  |  |
| Pots et al. (2016); the Netherlands | 236 adults with mild to moderate depressive symptoms; 46.9 years; 75.8% | - IG: 9 weekly modules of a guided web-based self-help ACT program over a 12-week intervention period, involving experiential exercises, metaphors, text messages, tailored stories, and daily 10-15 minute mindfulness exercises provided on audio files, with personal feedback and support by a therapist via e-mail (*n* = 82)  - CG1: 9 weekly modules of a guided web-based expressive writing program over a 12-week intervention period, involving writing about negative experiences, emotion regulation and reappraisal of emotions, writing about positive experiences and self-management for preventive purposes, with personal feedback and support by a therapist via email (*n* = 67)  - CG2: Waitlist control (*n* = 87) | Psychological flexibility/acceptance (AAQ-II) and mindfulness (FFMQ-short form); Pretest, posttest, 3-month F/U, and 9-month F/U; A significant improvement on AAQ-II in IG compared to CG1 and CG2 over time (*p* < 0.05) | Low |
|  |  |  |  |  |
| Räsänen et al. (2016); Finland | 68 university students experiencing psychological distress; 24.3 years; 85.3% | - IG: 7 weekly modules of a guided online ACT program, involving 2 face-to-face meetings with an assigned coach, online modules during a 5-week period (eg, self-help related text, weekly well-being tasks, experiential exercises in audio and video format, relevant metaphors, and case study vignettes), and personal weekly written feedback and support from trained student coaches via the website (*n* = 33)  - CG: Waitlist control (*n* = 35) | Psychological flexibility/ acceptance (AAQ-II) and mindfulness (FFMQ); Pretest, posttest, and 12-month F/U; A significant improvement on FFMQ in IG compared to CG over time (*p* < 0.05) | Unclear |
|  |  |  |  |  |
| Sagon et al. (2018); USA | 103 first-year college students; 18.2 years; 71.8% | - IG: An online version of an ACT workshop offered through Blackboard throughout the semester, involving four 10–15-minute narrated PowerPoint slides, audio recordings of guided mindfulness practices, 2 spaces for journal writing, and homework assignments (*n* = 52)  - CG: Waitlist control (*n* = 51) | Acceptance (Acceptance subscale of the PHLMS); Pretest and posttest; No significant between-group differences on Acceptance subscale of the PHLMS over time | Unclear |
|  |  |  |  |  |
| Sairanen et al. (2019); Sweden | 74 parents of children with chronic conditions (type 1 diabetes: 43.2%) experiencing significant burnout symptoms; 42.7 years; 81.1% | - IG: 5 modules of a guided ACT web intervention over a 10-week period, involving text, videos, exercises with MP3 audio files, questionnaires, homework assignments, a discussion forum, and a free-form diary, with written feedback from an assigned coach and an e-mail reminder (*n* = 37)  - CG: Waitlist control (*n* = 37) | Psychological flexibility/acceptance (AAQ-II), mindfulness (FFMQ), and cognitive fusion (CFQ); Pretest, posttest, and 4-month F/U; A significant improvement on FFMQ in IG compared to CG over time (*p* < 0.05) | Unclear |
|  |  |  |  |  |
| Scott et al. (2018); UK | 63 adult outpatients having chronic pain of at least 3 months duration and clinically significant pain, pain-related disability, and distress; 45.5 years; 63.5% | - IG: 8 guided online ACT sessions involving 8- to 27-minute videos with individualized written feedback from a therapist and reminders over a 10- to 12-week period that started with a 30- to 45-minute face-to-face or telephone session with the therapist and ended with a final face-to-face or telephone session (*n* = 31)  - CG: Usual care (*n* = 32) | Chronic pain acceptance (CPAQ-8) and valued living (CAQ-8); Pretest, posttest, and 6-month F/U; A large effect of IG on CPAQ-8 compared to CG at posttest | Low |
|  |  |  |  |  |
| Scott et al. (2021); UK | 38 adults with HIV, painful peripheral neuropathy, and at least moderate depressive symptoms; 55.9 years; 23.7% | - IG: 12 guided online ACT sessions (45–60 minutes each) over 8 weeks, involving brief videos and audio recordings which provided information about pain and guided participants through metaphors, experiential exercises, mindfulness practice, values clarification, and goal setting, with individualized feedback through written messages, brief telephone calls (10–30 minutes) or both and phone calls for support (*n* = 25)  - CG: Waitlist control (*n* = 13) | Chronic pain acceptance (CPAQ-8); Pretest, posttest, and 3-month F/U; Small to medium effects of IG on CPAQ-8 compared to CG at posttest and F/U | Low |
|  |  |  |  |  |
| Simister et al. (2018); Canada | 61 adults with fibromyalgia; 39.7 years; 95% | - IG: 7 modules of an online ACT program over an 8-week period, involving MP3 audio recordings, videos, metaphors, experiential exercises, introductory and recurring vignettes, and experiential homework exercises, with weekly email reminders and written feedback by the first author for clarification and positive reinforcement (*n* = 33)  - CG: Usual care (*n* = 34) | Chronic pain acceptance (CPAQ-Revised), mindfulness (FFMQ), cognitive fusion (CFQ), and valued living (VLQ); Pretest, posttest, and 3-month F/U; Significant improvements on CPAQ-Revised (*p* < 0.05), CFQ (*p* < 0.01) , and VLQ (*p* < 0.05) in IG compared to CG over time | Low |
|  |  |  |  |  |
| Trompetter et al. (2015); the Netherlands | 238 adults with chronic pain; 54.9 years; 76% | - IG: 9 modules of an internet-delivered, guided self-help ACT program over a 9- to 12-week period, involving text, experiential exercises, metaphors, downloadable 10- to 15-minute mindfulness exercises, and daily mindfulness practice, with personal feedback and support by a therapist via email (*n* = 82)  - CG1: 9 modules of an internet-based expressive writing program over a 9- to 12-week period (an active control), involving psychoeducation about emotions and emotion regulation related to the pain experience, writing about specific negative or positive experiences, assignments of writing at least 3 times a week for 15 minutes, and keeping a personal diary, with personal feedback and support by a therapist via email (*n* = 79)  - CG2: Waitlist control (*n* = 77) | Psychological inflexibility (PIPS), mindfulness (FFMQ-SF), and valued living (ELS); Pretest, posttest, and 3-month F/U; A significant improvement on PIPS in IG compared to CG1 (*p* < 0.05) and CG2 (*p* < 0.001) over time | Unclear |
|  |  |  |  |  |
| Twohig et al. (2021); USA | 28 adolescents diagnosed with trichotillomania; 13.9 years; 67.9% | - IG: 10 sessions (50 minutes per session) of an ACT-enhanced behavior therapy delivered via Zoom videoconferencing and homework assignments between sessions (*n* = 14)  - CG: Waitlist control (*n* = 14) | Acceptance for Trichotillomania (AAQ-TTM); Pretest and posttest; A medium effect of IG on AAQ-TTM compared to CG at posttest | Unclear |
|  |  |  |  |  |
| van Aubel et al. (2020); Belgium | 55 emerging adults (aged 16 to 25 years) with subthreshold depressive and/or psychotic complaints; 21 years; 72.7% | - IG: Blended care, involving 5 weekly face-to-face group-based ACT sessions with a trained therapist (1.5 hours per session) and daily use of the ACT-DL app between sessions for 6 weeks, providing assessment asking about current mood, context, and activities at 8 semi-random moments throughout the day for 3 subsequent days per week and options to do ACT skill practices (*n* = 27)  - CG: 5 weekly face-to-face group based sessions, involving watching 1 of 5 documentaries on biography, crime, history, nature, or art and discussing several questions about the documentary (eg, what they thought was the main message and how they could apply this message to their lives) (*n* = 28) | Psychological flexibility (FIT-60); Pretest, posttest, 6-month F/U, and 12-month F/U; No significant between-group differences on FIT-60 over time | Low |
|  |  |  |  |  |
| Viskovich & Pakenham (2020); Australia | 1162 university students; 26.9 years; 67.8% | - IG: 4 weekly modules (30-45 minutes per module) of a web-based ACT program, involving animated presentations, video clips, audio files, and written exercises, with nonpersonalized reminder emails to prompt program engagement and standard SMS text messages or emails to reinforce program content (*n* = 596)  - CG: Waitlist control (*n* = 566) | Psychological flexibility/acceptance (AAQ-II), cognitive fusion (CFQ), valued living (ELS), and mindfulness (MAAS); Pretest, posttest, and 3-month F/U; Significant improvements on AAQ-II, CFQ, ELS, and MAAS in IG compared to CG over time (*p* < 0.001) | Unclear |
|  |  |  |  |  |
| Weineland et al. (2012); Sweden | 39 adults who underwent bariatric surgery; 43.1 years; 89.7% | - IG: An ACT program, involving two 1.5-hour face-to-face sessions, 6 weekly internet-delivered self-help ACT modules (texts, mindfulness audio files, written exercises, and audio visual animations), and a 30-minute support session weekly over the telephone (*n* = 19)  - CG: Usual care (*n* = 20) | Acceptance for weight (AAQ-W); Pretest and posttest; A significant improvement on AAQ-W in IG compared to CG over time (*p* < 0.01) | Unclear |

Abbreviations: AAQ, Acceptance and Action Questionnaire; AAQ-TTM, Acceptance and Action Questionnaire for Trichotillomania; AAQ-W, Acceptance and Action Questionnaire for Weight; ACT, acceptance and commitment therapy; AFQ-Y, Avoidance and Fusion Questionnaire for Youth; AIS, Avoidance and Inflexibility Scale; CAQ, Committed action questionnaire; CBT, cognitive behavioral therapy; CFQ, Cognitive Fusion Questionnaire; CG, control group; CPAQ, Chronic Pain Acceptance Questionnaire; CQS, Commitment to Quitting Scale; ELS, Engaged Living Scale; FFMQ, Facet Mindfulness Questionnaire; FIT-60, Flexibility Index Test; F/U, follow-up; HIV, human immunodeficiency virus; ICQ-P, Illness Cognition Questionnaire for Parents; IG, intervention group; MAAS, Mindful Attention Awareness Scale; PHLMS, Philadelphia Mindfulness Scale; PIPS, Psychological Inflexibility in Pain Scale; PPFQ, Parental Psychological Flexibility Questionnaire; PVQ, Personal Values Questionnaire; RoB: risk of bias; TAQ, Tinnitus Acceptance Questionnaire; VLQ, Valued Living Questionnaire; VQ, Valuing Questionnaire; WAAQ, Work-related Acceptance and Action Questionnaire.
