# Supplementary figures and images for: The Effects of Internet-Based Acceptance and Commitment Therapy on Process Measures: Systematic Review and Meta-analysis
Source: J Med Internet Res. 2022 Aug 30;24(8):e39182. doi: 10.2196/39182 (PMC9472046; doi:10.2196/39182)

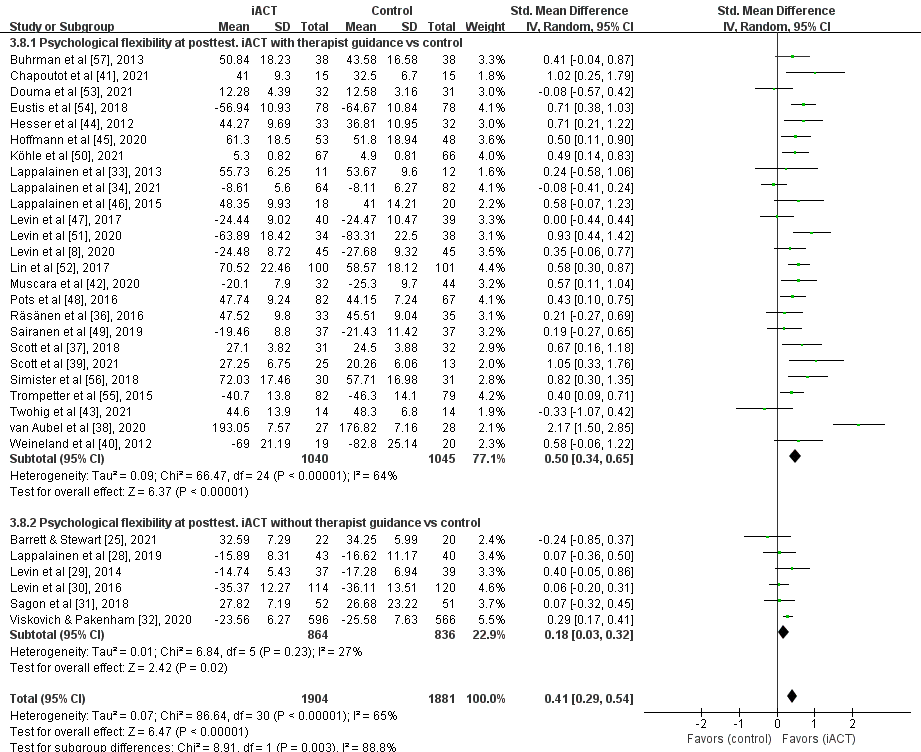

Supplement: Multimedia Appendix 3 [file jmir_v24i8e39182_app3.docx]

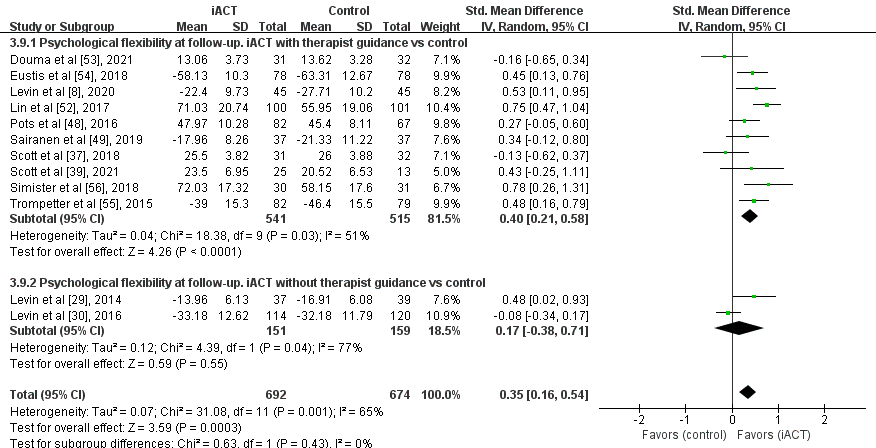

Supplement: Multimedia Appendix 4 [file jmir_v24i8e39182_app4.docx]

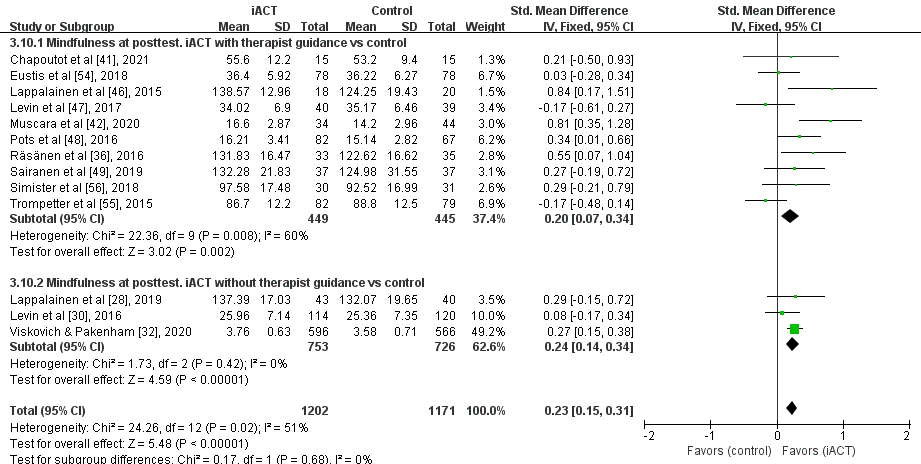

Supplement: Multimedia Appendix 5 [file jmir_v24i8e39182_app5.docx]

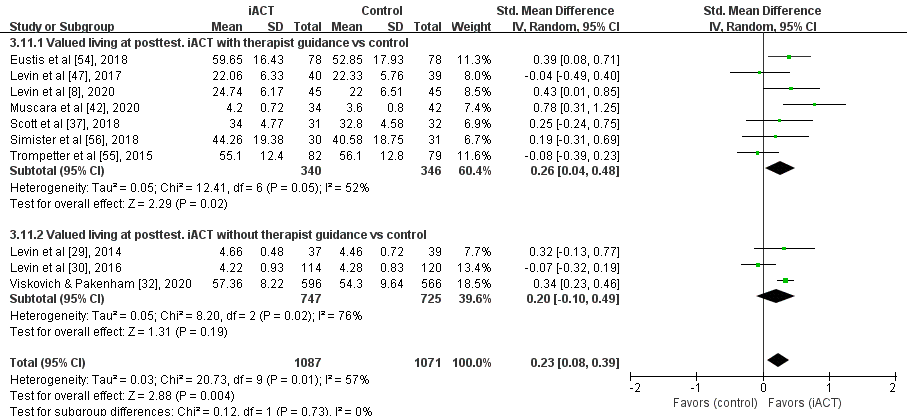

Supplement: Multimedia Appendix 6 [file jmir_v24i8e39182_app6.docx]

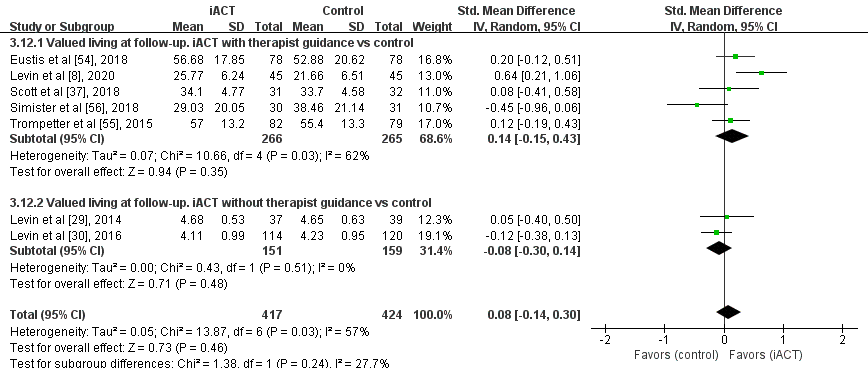

Supplement: Multimedia Appendix 7 [file jmir_v24i8e39182_app7.docx]

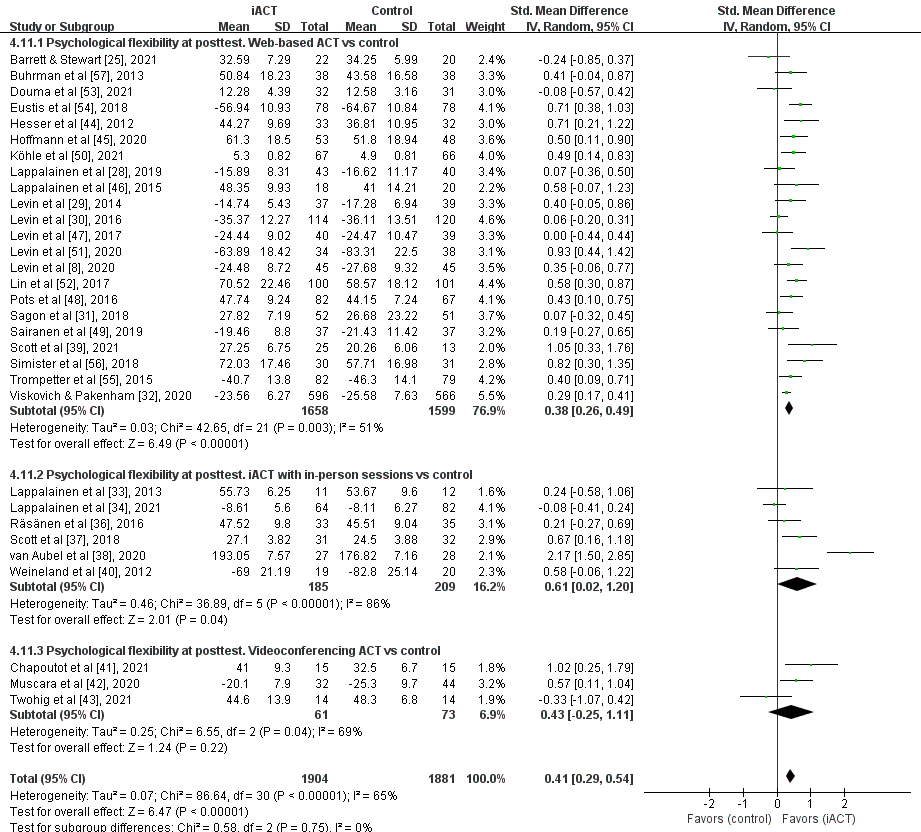

Supplement: Multimedia Appendix 8 [file jmir_v24i8e39182_app8.docx]

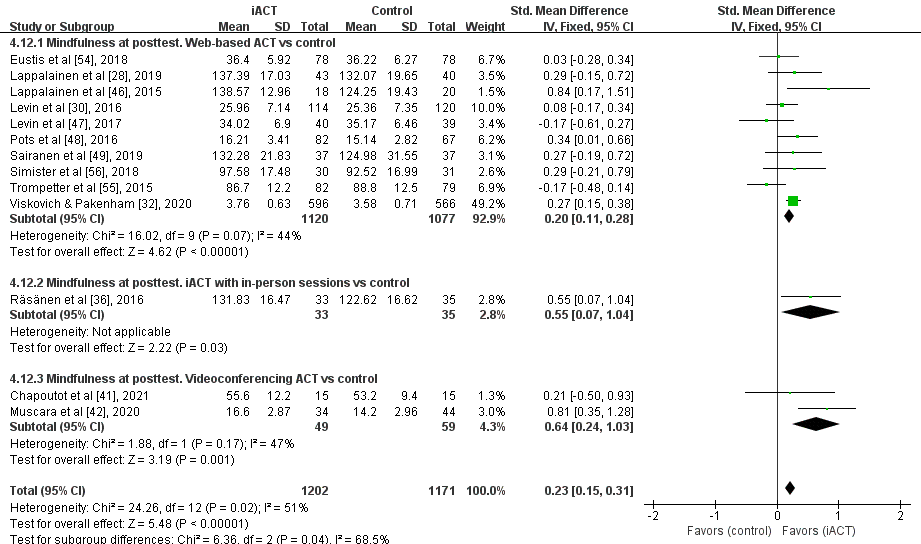

Supplement: Multimedia Appendix 9 [file jmir_v24i8e39182_app9.docx]

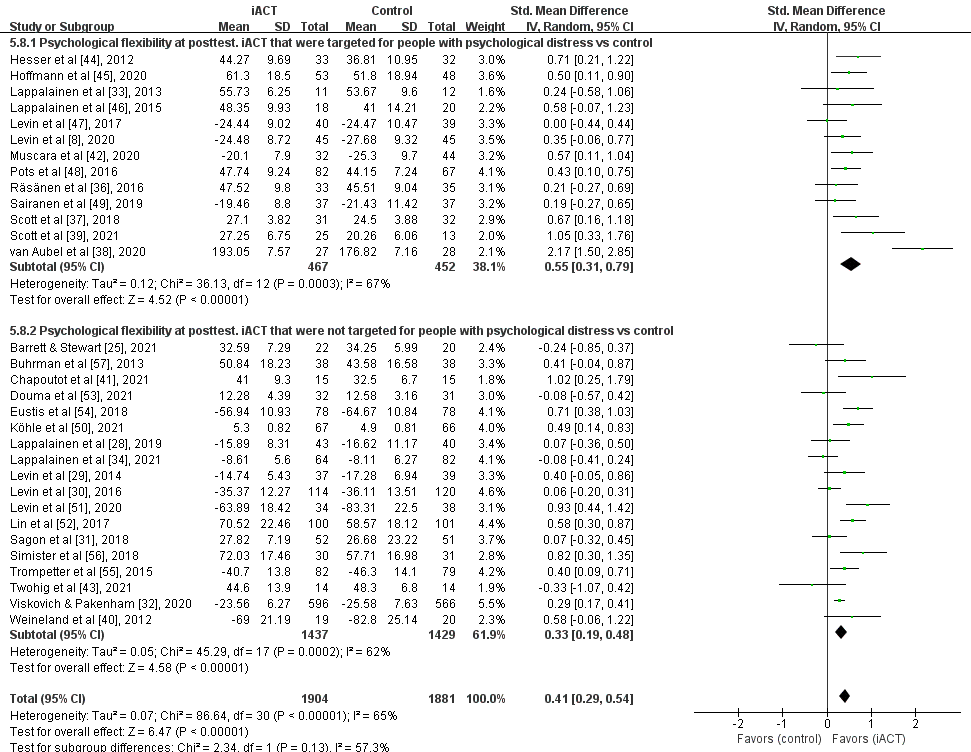

Supplement: Multimedia Appendix 10 [file jmir_v24i8e39182_app10.docx]

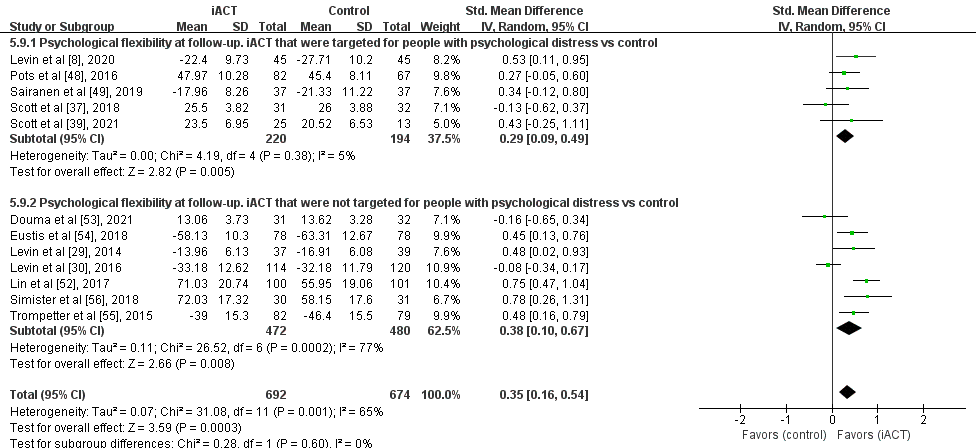

Supplement: Multimedia Appendix 11 [file jmir_v24i8e39182_app11.docx]

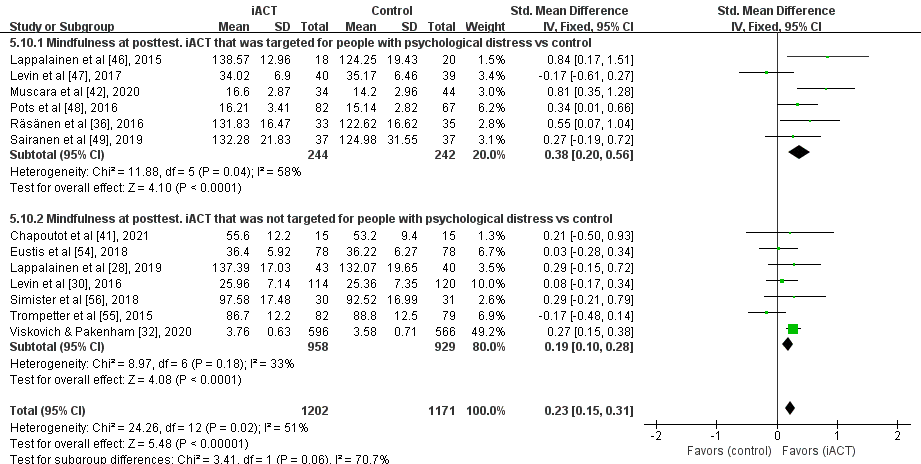

Supplement: Multimedia Appendix 12 [file jmir_v24i8e39182_app12.docx]

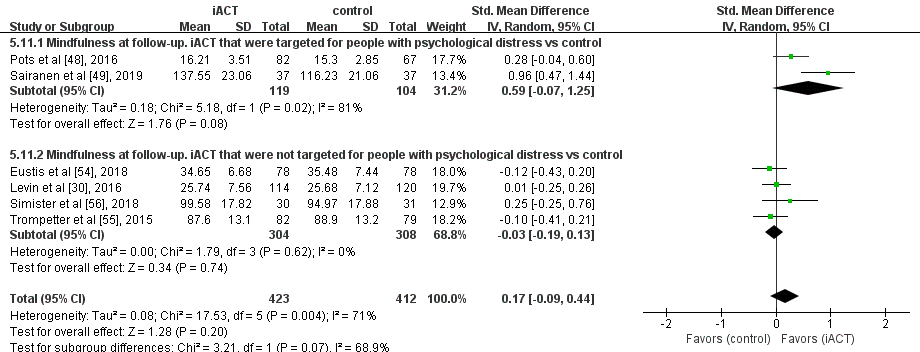

Supplement: Multimedia Appendix 13 [file jmir_v24i8e39182_app13.docx]

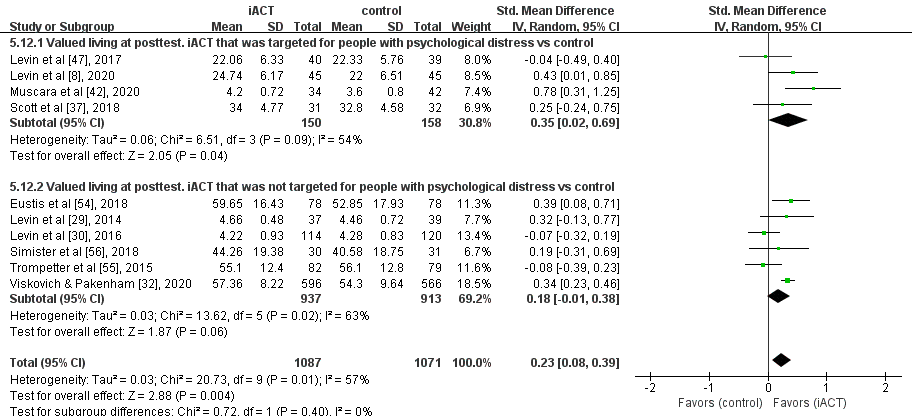

Supplement: Multimedia Appendix 14 [file jmir_v24i8e39182_app14.docx]

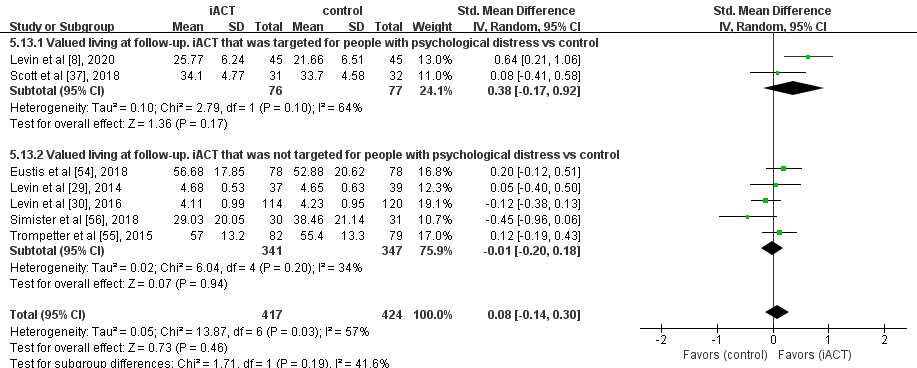

Supplement: Multimedia Appendix 15 [file jmir_v24i8e39182_app15.docx]

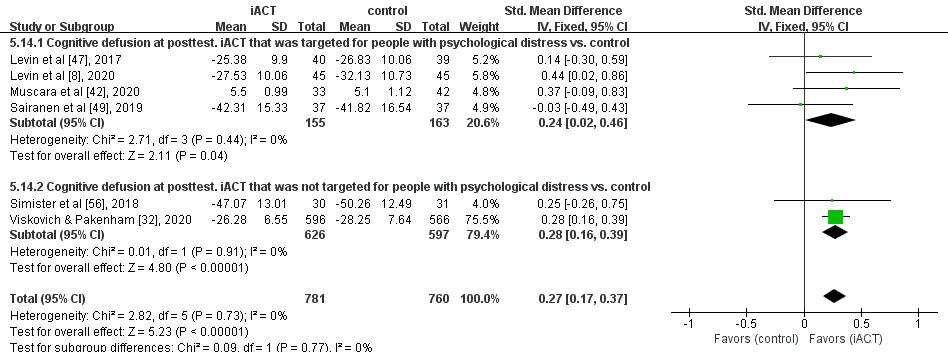

Supplement: Multimedia Appendix 16 [file jmir_v24i8e39182_app16.docx]

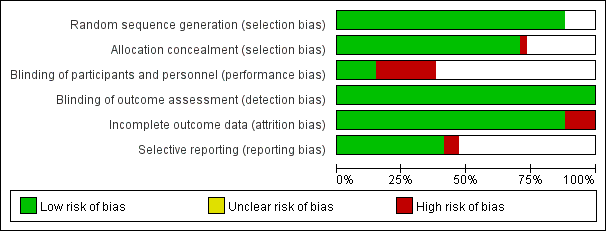

Supplement: Multimedia Appendix 19 [file jmir_v24i8e39182_app19.docx]

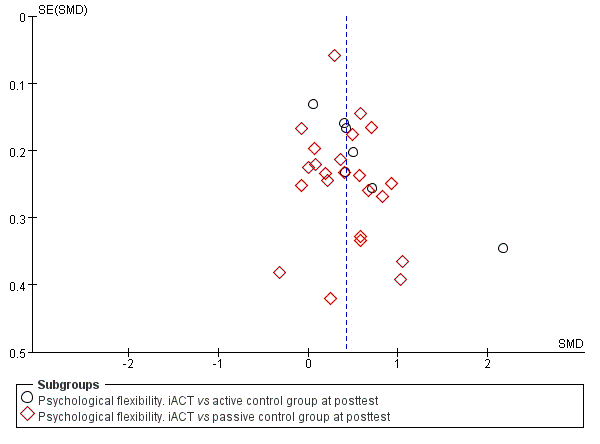


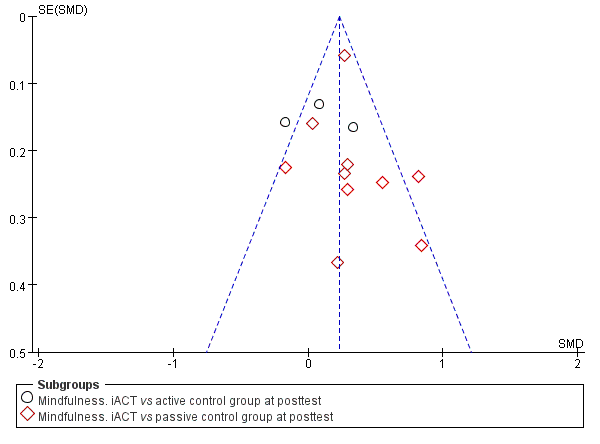

Supplement: Multimedia Appendix 20 [file jmir_v24i8e39182_app20.docx]
