# Supplementary material for: The Effects of Internet-Based Acceptance and Commitment Therapy on Process Measures: Systematic Review and Meta-analysis
Source: J Med Internet Res. 2022 Aug 30;24(8):e39182. doi: 10.2196/39182 (PMC9472046; doi:10.2196/39182)
Supplement: Multimedia Appendix 17 [file jmir_v24i8e39182_app17.docx]

**Risk of Bias (RoB) of the Included Studies**

| Reference | Random sequence generation | Allocation concealment | Blinding of participants and personnel | Blinding of outcome assessment | Incomplete outcome data | Selective reporting | Overall RoB |
| --- | --- | --- | --- | --- | --- | --- | --- |
| Barrett & Stewart (2021) | Low | Unclear | Unclear | Low | Low | Unclear | Unclear |
| Bricker et al. (2013) | Low | Low | Low | Low | High | High | High |
| Buhrman et al. (2013) | Low | Low | High | Low | Low | Low | Low |
| Chapoutot et al. (2021) | Low | Unclear | Unclear | Low | Low | Unclear | Unclear |
| Douma et al. (2021) | Low | Low | High | Low | High | Low | High |
| Eustis et al. (2018) | Low | High | Unclear | Low | Low | Unclear | High |
| Heffner et al. (2020) | Unclear | Unclear | High | Low | Low | High | High |
| Hesser et al. (2012) | Low | Low | Unclear | Low | Low | Unclear | Unclear |
| Hoffmann et al. (2020) | Low | Low | Unclear | Low | Low | Low | Low |
| Köhle et al. (2021) | Low | Low | High | Low | Low | Low | Low |
| Lappalainen et al. (2013) | Low | Unclear | High | Low | Low | Unclear | Unclear |
| Lappalainen et al. (2015) | Low | Low | Unclear | Low | Low | Unclear | Unclear |
| Lappalainen et al. (2019) | Low | Low | Unclear | Low | Low | Unclear | Unclear |
| Lappalainen et al. (2021) | Low | Low | Unclear | Low | Low | Unclear | Unclear |
| Levin et al. (2014) | Low | Unclear | Unclear | Low | Low | Unclear | Unclear |
| Levin et al. (2016) | Low | Low | Low | Low | Low | Unclear | Unclear |
| Levin et al. (2017) | Low | Low | High | Low | Low | Unclear | Unclear |
| Levin et al. (2020a) | Low | Low | Unclear | Low | Low | Unclear | Unclear |
| Levin et al. (2020b) | Unclear | Unclear | Unclear | Low | High | Low | High |
| Lin et al. (2017) | Low | Low | Unclear | Low | Low | Low | Low |
| Muscara et al. (2020) | Low | Low | Low | Low | High | Low | High |
| O'Connor et al. (2020) | Low | Low | Low | Low | Low | Low | Low |
| Pots et al. (2016) | Low | Low | Unclear | Low | Low | Low | Low |
| Räsänen et al. (2016) | Low | Low | Unclear | Low | Low | Unclear | Unclear |
| Sagon et al. (2018) | Unclear | Unclear | Unclear | Low | Low | Unclear | Unclear |
| Sairanen et al. (2019) | Low | Low | Unclear | Low | Low | Unclear | Unclear |
| Scott et al. (2018) | Low | Low | High | Low | Low | Low | Low |
| Scott et al. (2021) | Low | Low | High | Low | Low | Low | Low |
| Simister et al. (2018) | Low | Low | Unclear | Low | Low | Low | Low |
| Trompetter et al. (2015) | Low | Unclear | Unclear | Low | Low | Low | Unclear |
| Twohig et al. (2021) | Low | Low | Unclear | Low | Low | Unclear | Unclear |
| van Aubel et al. (2020) | Low | Low | Low | Low | Low | Low | Low |
| Viskovich & Pakenham (2020) | Low | Low | Unclear | Low | Low | Unclear | Unclear |
| Weineland et al. (2012) | Unclear | Unclear | Unclear | Low | Low | Unclear | Unclear |
