# Supplementary material for: The Effects of Internet-Based Acceptance and Commitment Therapy on Process Measures: Systematic Review and Meta-analysis
Source: J Med Internet Res. 2022 Aug 30;24(8):e39182. doi: 10.2196/39182 (PMC9472046; doi:10.2196/39182)
Supplement: Multimedia Appendix 18 [file jmir_v24i8e39182_app18.docx]

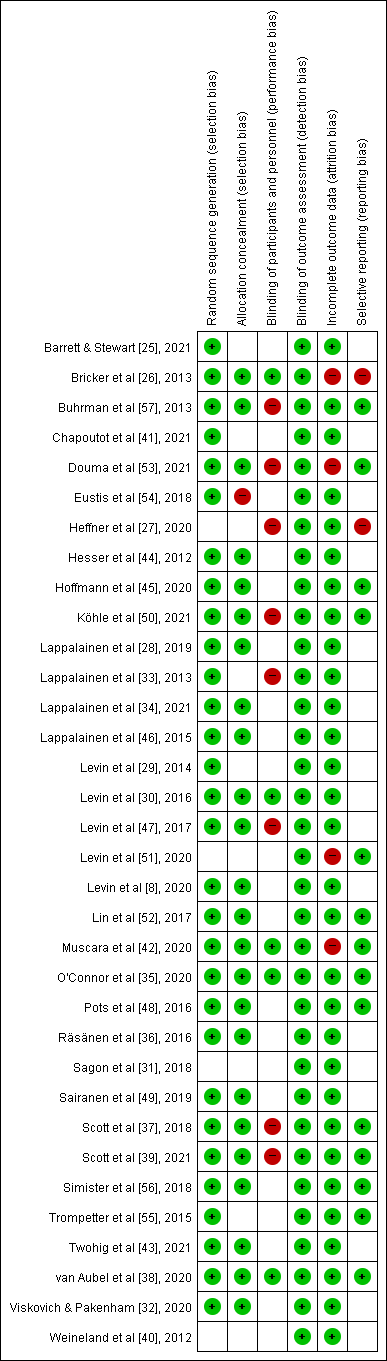


8. Levin ME, Krafft J, Hicks ET, Pierce B, Twohig MP. A randomized dismantling trial of the open and engaged components of acceptance and commitment therapy in an online intervention for distressed college students. Behav Res Ther 2020;126:103557. <https://doi.org/10.1016/j.brat.2020.103557>

25. Barrett, K., & Stewart, I. (2021). A preliminary comparison of the efficacy of online Acceptance and Commitment Therapy (ACT) and Cognitive Behavioural Therapy (CBT) stress management interventions for social and healthcare workers. Health Soc Care Community 2021;29(1):113-126. <https://doi.org/10.1111/hsc.13074>

26. Bricker, J., Wyszynski, C., Comstock, B., & Heffner, J. L. (2013). Pilot randomized controlled trial of web-based acceptance and commitment therapy for smoking cessation. Nicotine Tob Res 2013;15(10):1756-1764. <https://doi.org/10.1093/ntr/ntt056>

27. Heffner JL., Kelly MM, Waxmonsky J, Mattocks K, Serfozo E, Bricker JB, et al. Pilot randomized controlled trial of web-delivered acceptance and commitment therapy versus smokefree.gov for smokers with bipolar disorder. Nicotine Tob Res 2020;22(9):1543-1552. <https://doi.org/10.1093/ntr/ntz242>

28. Lappalainen P, Langrial S, Oinas-Kukkonen H, Muotka J, Lappalainen R. ACT for sleep - Internet-delivered self-help ACT for sub-clinical and clinical insomnia: A randomized controlled trial. J Context Behav Sci 2019;12:119-127. <https://doi.org/http://dx.doi.org/10.1016/j.jcbs.2019.04.001>

29. Levin ME, Pistorello J, Seeley JR, Hayes SC. Feasibility of a prototype web-based acceptance and commitment therapy prevention program for college students. J Am Coll Health 2014;62(1):20-30. <https://doi.org/10.1080/07448481.2013.843533>

30. Levin ME, Hayes SC, Pistorello J, Seeley JR. Web-based self-help for preventing mental health problems in universities: Comparing acceptance and commitment training to mental health education. J Clin Psychol 2016;72(3):207-225. <https://doi.org/10.1002/jclp.22254>

31. Sagon AL, Danitz SB, Suvak MK, Orsillo SM. (2018). The Mindful Way through the Semester: Evaluating the feasibility of delivering an acceptance-based behavioral program online. J Context Behav Sci 2018;9:36-44. <https://doi.org/10.1016/j.jcbs.2018.06.004>

32. Viskovich S, Pakenham KI. Randomized controlled trial of a web-based Acceptance and Commitment Therapy (ACT) program to promote mental health in university students. J Clin Psychol 2020;76(6):929-951. <https://doi.org/10.1002/jclp.22848>

33. Lappalainen, P, Kaipainen, K, Lappalainen, R, Hoffrén, H, Myllymäki, T, Kinnunen ML, et al. (2013). Feasibility of a personal health technology-based psychological intervention for men with stress and mood problems: Randomized controlled pilot trial. JMIR Res Protoc 2013;2(1):e1. <https://doi.org/10.2196/resprot.2389>

34. Lappalainen R, Lappalainen P, Puolakanaho A, Hirvonen R, Eklund K, Ahonen T, et al. (2021). The Youth Compass - the effectiveness of an online acceptance and commitment therapy program to promote adolescent mental health: A randomized controlled trial. J Context Behav Sci 2021;20:1-12. <https://doi.org/10.1016/j.jcbs.2021.01.007>

35. O'Connor M, Whelan R, Bricker J, McHugh L. (2020). Randomized controlled trial of a smartphone application as an adjunct to acceptance and commitment therapy for smoking cessation. Behav Ther 2020;51(1):162-177. <https://doi.org/10.1016/j.beth.2019.06.003>

36. Räsänen P, Lappalainen P, Muotka J, Tolvanen A, Lappalainen R. (2016). An online guided ACT intervention for enhancing the psychological wellbeing of university students: A randomized controlled clinical trial. Behav Res Ther 2016;78:30-42. <https://doi.org/10.1016/j.brat.2016.01.001>

37. Scott W, Chilcot J, Guildford B, Daly‐Eichenhardt A, McCracken LM. Feasibility randomized‐controlled trial of online Acceptance and Commitment Therapy for patients with complex chronic pain in the United Kingdom. Eur J Pain 2018;22(8):1473-1484. <https://doi.org/10.1002/ejp.1236>

38. Van Aubel E, Bakker JM, Batink T, Michielse S, Goossens L, Lange I, et al. (2020). Blended care in the treatment of subthreshold symptoms of depression and psychosis in emerging adults: A randomised controlled trial of Acceptance and Commitment Therapy in Daily-Life (ACT-DL). Behav Res Ther 2020;128:103592. <https://doi.org/10.1016/j.brat.2020.103592>

39. Scott W, Guildford BJ, Badenoch J, Driscoll E, Chilcot J, Norton S, et al. Feasibility randomized‐controlled trial of online acceptance and commitment therapy for painful peripheral neuropathy in people living with HIV: The open study. 2021;25(7):1493-1507 <https://doi.org/http://dx.doi.org/10.1002/ejp.1762>

40. Weineland S, Arvidsson D, Kakoulidis TP, Dahl J. Acceptance and commitment therapy for bariatric surgery patients, a pilot RCT. Obes Res Clin Pract 2012;6(1):e1-e90. <https://doi.org/10.1016/j.orcp.2011.04.004>

41. Chapoutot M, Peter-Derex L, Schoendorff B, Faivre T, Bastuji H, Putois B. Telehealth-delivered CBT-I programme enhanced by acceptance and commitment therapy for insomnia and hypnotic dependence: A pilot randomized controlled trial. J Sleep Res 2021;30(1):e13199. <https://doi.org/10.1111/jsr.13199>

42. Muscara F, McCarthy MC, Rayner M, Nicholson JM, Dimovski A, McMillan L, et al. Effect of a videoconference-based online group intervention for traumatic stress in parents of children with life-threatening illness: A randomized clinical trial. JAMA Netw Open 2020;3(7):e208507. <https://doi.org/10.1001/jamanetworkopen.2020.8507>

43. Twohig MP, Petersen JM, Fruge J, Ong CW, Barney JL, Krafft J, et al. A pilot randomized controlled trial of online-delivered ACT-enhanced behavior therapy for trichotillomania in adolescents. Cogn Behav Pract 2021;28(4):653-668. <https://doi.org/10.1016/j.cbpra.2021.01.004>

44. Hesser H, Gustafsson T, Lundén C, Henrikson O, Fattahi K, Johnsson E., et al. A randomized controlled trial of internet-delivered cognitive behavior therapy and acceptance and commitment therapy in the treatment of tinnitus. J Consult Clin Psychol 2012;80(4):649-661. <https://doi.org/http://dx.doi.org/10.1037/a0027021>

45. Hoffmann D, Rask CU, Hedman-Lagerlöf E, Jensen JS, Frostholm L. Efficacy of internet-delivered acceptance and commitment therapy for severe health anxiety: Results from a randomized, controlled trial. Psychol Med 2021;51(15):2685-2695. <https://doi.org/10.1017/s0033291720001312>

46. Lappalainen P, Langrial S, Oinas-Kukkonen H, Tolvanen A, Lappalainen R. Web-based acceptance and commitment therapy for depressive symptoms with minimal support: A randomized controlled trial. Behav Modif 2015;39(6):805-834. <https://doi.org/10.1177/0145445515598142>

47. Levin ME, Haeger JA, Pierce BG, Twohig MP. Web-based acceptance and commitment therapy for mental health problems in college students: A randomized controlled trial. Behav Modif 2017;41(1):141-162. <https://doi.org/10.1177/0145445516659645>

48. Pots WTM., Fledderus M, Meulenbeek PAM., ten Klooster PM, Schreurs KMG, Bohlmeijer ET. Acceptance and commitment therapy as a web-based intervention for depressive symptoms: Randomised controlled trial. Br J Psychiatry 2016;208(1):69-77. <https://doi.org/http://dx.doi.org/10.1192/bjp.bp.114.146068>

49. Sairanen E, Lappalainen R, Lappalainen P, Kaipainen K, Carlstedt F, Anclair M, et al. (2019). Effectiveness of a web-based Acceptance and Commitment Therapy intervention for wellbeing of parents whose children have chronic conditions: A randomized controlled trial. J Context Behav Sci 2019;13:94-102. <https://doi.org/http://dx.doi.org/10.1016/j.jcbs.2019.07.004>

50. Köhle N, Drossaert CHC, Ten Klooster PM, et al. Web-based self-help intervention for partners of cancer patients based on acceptance and commitment therapy and self-compassion training: A randomized controlled trial with automated versus personal feedback. Support Care Cancer 2021;29:5115-5125. <https://doi.org/10.1007/s00520-021-06051-w>

51. Levin ME, Petersen JM, Durward C, et al. A randomized controlled trial of online acceptance and commitment therapy to improve diet and physical activity among adults who are overweight/obese. Transl Behav Med 2020;11:1216-1225. <https://doi.org/10.1093/tbm/ibaa123>

52. Lin J, Paganini S, Sander L, et al. An internet-based intervention for chronic pain: A three-arm randomized controlled study of the effectiveness of guided and unguided acceptance and commitment therapy. Dtsch Arztebl Int 2017;114:681-688. <https://doi.org/10.3238/arztebl.2017.0681>

53. Douma M, Maurice-Stam H, Gorter B, et al. Online psychosocial group intervention for parents: Positive effects on anxiety and depression. J Pediatr Psychol 2021;46:123-134. <https://doi.org/10.1093/jpepsy/jsaa102>

54. Eustis EH, Hayes-Skelton SA, Orsillo SM, Roemer L. Surviving and thriving during stress: A randomized clinical trial comparing a brief web-based therapist-assisted acceptance-based behavioral intervention versus waitlist control for college students. Behav. Ther. 2018;49:889-903.<https://doi.org/10.1016/j.beth.2018.05.009>

55. Trompetter H, Bohlmeijer E, Veehof M, Schreurs K. Internet-based guided self-help intervention for chronic pain based on Acceptance and Commitment Therapy: A randomized controlled trial. J. Behav. Med 2015;38:66-80. <https://doi.org/10.1007/s10865-014-9579-0>

56. Simister HD, Tkachuk GA, Shay BL, Vincent N, Pear JJ, Skrabek RQ. Randomized controlled trial of online acceptance and commitment therapy for fibromyalgia. J Pain. 2018;19:741-753. <https://doi.org/10.1016/j.jpain.2018.02.004>

57. Buhrman M, Skoglund A, Husell J, et al. Guided internet-delivered acceptance and commitment therapy for chronic pain patients: A randomized controlled trial. Behav Res Ther. 2013;51:307-15. <https://doi.org/10.1016/j.brat.2013.02.010>
